# Transcriptomic analysis and high throughput functional characterization of human induced pluripotent stem cell derived sensory neurons^☆^

**DOI:** 10.1016/j.ynpai.2026.100216

**Published:** 2026-05-01

**Authors:** Vincent Truong, Jackson Brougher, Tim Strassmaier, Yi-Ling Lu, Dale George, Theodore J. Price, Alison Obergrussberger, Rodolfo J. Haedo, Niels Fertig, Patrick Walsh

**Affiliations:** a Anatomic Incorporated, 2112 Broadway Street NE #135, Minneapolis, MN 55413, United States; b Doloromics, 3515 Edison Way, Suite C, Menlo Park, CA 94025, United States; c Nanion Technologies Inc., 1 Naylon Place Livingston, NJ 07039, United States; d University of Texas at Dallas, Center for Advanced Pain Studies and Department of Neuroscience, 800 W Campbell Rd, Richardson, TX 75080, United States; e Nanion Technologies GmbH, Ganghoferstr. 70A, 80339 Munich, Germany

**Keywords:** hiPSC, APC, Ion channels, RNA sequencing, Sensory neurons, DRG

## Abstract

Peripheral sensory neurons are a primary effector in pain neurotransmission, and have become a useful cellular model for the study of pain. While rodent tissue has historically served as a source of these neurons, it has become increasingly clear that pain mechanisms in rodents and humans are substantially divergent. Sensory neurons harvested from cadaveric human tissue serve as a superior translational model for studying pain mechanisms, however their relative paucity limits their widespread utility. Theoretically, sensory neurons manufactured from human induced pluripotent stem cells (hiPSCs) could help bridge this translational gap given their relative abundance and potential similarity to primary human tissue. However, hiPSC-derived sensory neurons manufactured with the most common methodologies correlate poorly to human tissue both transcriptionally and functionally. In the present work, we compare a population of hiPSC-derived sensory neurons (hiSNs) generated using an accelerated directed differentiation method to previously published datasets and find this population to more closely resemble human primary dorsal root ganglia transcriptionally. Furthermore, we evaluate the heterogeneity of this novel population via single nucleus RNA sequencing and find it resembles specific nociceptor and mechanoreceptor subsets found in vivo. Finally, we assay the functionality of this population with high throughput automated patch clamp electrophysiology recordings of voltage-gated TTX-sensitive and TTX-resistant sodium (Na_v_), potassium channels (K_v_), ligand-gated ionotropic GABA and P2X receptors, and action potentials. Overall, we find this population of hiSNs demonstrates measurable expression and functional activity across multiple pain-relevant targets, making it suitable for interrogating diverse pain mechanisms on a fully humanized platform.

## Highlights

• Novel hPSC-derived sensory neurons closely matching human dorsal root ganglia.
• snRNA-seq shows this population mirrors specific in vivo nociceptor and mechanoreceptor subsets.
• Automated patch clamp confirms expected voltage-, ligand- and mechano-gated currents.
• Potential tool for drug discovery in pain research.

## Introduction

Pain is a complex and debilitating condition that will affect nearly every living human throughout their lifetime. Despite significant advances in our understanding of the molecular and cellular mechanisms underlying pain, the development of effective pain therapeutics that are devoid of major side-effects or toxicities has been challenging. Part of this difficulty stems from the limited availability of human models for both pain drug discovery and fundamental, mechanism-based research (Renthal, 2021). Sensory neurons, a key component of the peripheral nervous system, play a crucial role in conveying nociceptive signals from the body to the spinal cord and brain where pain is ultimately perceived (Basbaum, 2009). Traditional preclinical models predominantly utilize primary rodent dorsal root ganglion (DRG) cells, the overexpression of ion channel targets in human embryonic kidney (HEK) or Chinese hamster ovary (CHO) cell lines, or ideally human DRG − which is a scarce resource for most laboratories (Renthal, 2021). hiPSC-derived sensory neurons are a scalable and pertinent alternative, offering a viable and efficient approach for modeling pain in high-throughput drug discovery efforts (Schrenk-Siemens et al., 2018).

Multiple human iPSC sensory neuron protocols exist in the literature based on two traditional methods. The first are directed differentiation methods replicating nervous system development using the classical dual SMAD inhibition based neural induction (Chambers, 2009) or nocispheres (Deng, 2023), patterning to a neural crest intermediate, and then terminal differentiation to sensory neurons with varying levels of efficiencies (Deng, 2023, Clark, 2021, Chambers, 2012, Lampert, 2020). Alternatively, there are protocols for the overexpression of cell-fate determining transcription factor programming to generate mechanoreceptors (Nickolls, 2020) or nociceptors (Plumbly, 2022). A recently described method of directed differentiation through an intermediate primal ectoderm population (Walsh, 2020) has been shown to reproducibly generate hiSNs (commercially available as RealDRG) (Li, 2021, Jiang, 2023) where voltage-gated sodium (Jami, 2023, Kalia, 2023) and Piezo2 (Romero, 2023, Gabrielle, et al., 2024) channels have been functionally validated − along with their utility for optimization of cryopreservation (Li, 2021) and numerous other applications (Jiang, 2023, Geramifard and Novel, 2022, Zurek, 2023, Pollard, 2023, Didier, 2023, Ehsanian, 2021). A comparison of representative hiPSC-derived sensory neuron differentiation strategies is provided in Supplementary Table 1, highlighting key differences in induction approach, intermediates, and maturation timelines. In the studies described here, we have thoroughly characterized hiSNs throughout their maturation using RNA sequencing, as well as automated patch clamp electrophysiology (APC). This was done with the goal of understanding the utility of these neurons for drug screens and mechanistic studies, and to create a thorough resource for researchers who would use these neurons for these or other purposes. We have bulk RNA sequenced hiSNs through four weeks of maturation and compared the expression to both primary hDRG (Ray, 2018, Tavares-Ferreira, 2022) and other hiPSC-derived sensory neurons. Using single nuclei sequencing, we also look at how maturation develops through the first two weeks to determine what sensory neuron sub-type hiSNs are most similar to in hDRG. Finally, we sought to functionally validate a panel of expressed ion channel targets utilizing high-throughput APC electrophysiology.

## Results

### hiPSC-derived sensory neuron principal component analysis and comparison to hDRG

**Fig. 1:**
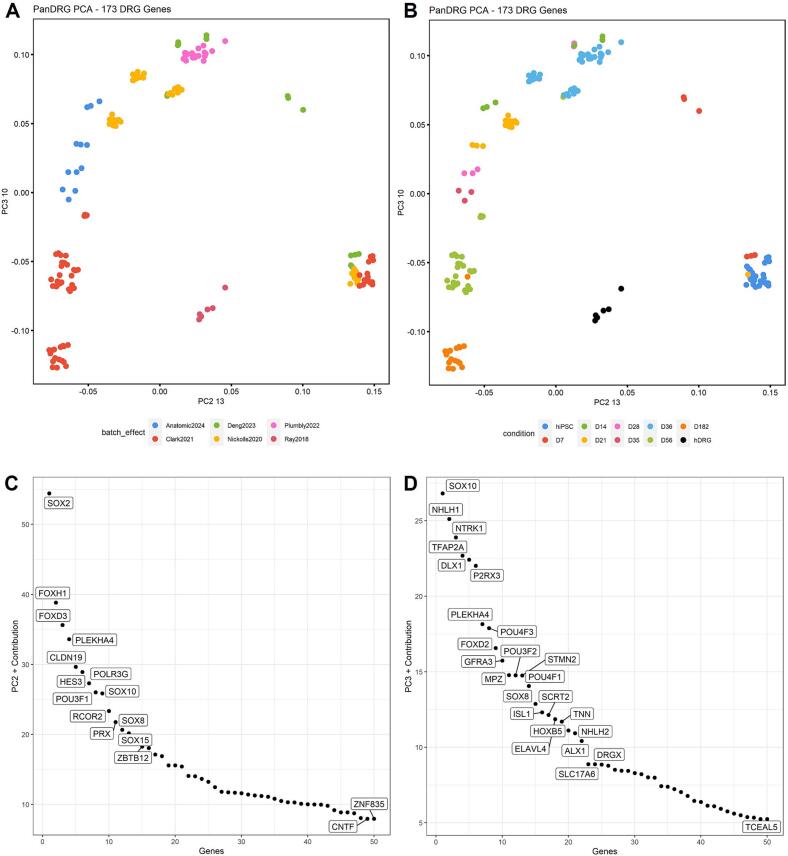
Transcriptomic analysis using a panel of 173 sensory neuron specific genes with data sets including primary human DRG, hiPSC-derived sensory neurons, and human induced pluripotent stem cells show differentiation and maturation trajectories. A) PCA plot of multiple RNA-seq data sets show distinct molecular signatures by protocol. B) PCA plot of multiple RNA-seq data sets show distinct molecular signatures at various timepoints. C) A list of genes that contribute to the variance of principal component 2 (PC2). D) A list of genes that contribute to the variance of principal component 3 (PC3).

To determine how the bulk transcriptome of hDRG and hiPSC-derived sensory neuron populations compare, a number of published datasets (Deng, 2023, Clark, 2021, Nickolls, 2020, Plumbly, 2022) were compiled from GEO and principal component analysis (PCA) was performed to explore the time course differentiation and maturation of hiPSC sensory neurons generated using various protocols. These datasets were compared to hiPSC lines (Deng, 2023, Clark, 2021, Nickolls, 2020), bulk sequenced hDRG, which contains a mix of neuronal subtypes, glia and other cells (Nickolls, 2020, Ray, 2018), and the novel differentiation method currently disclosed (Kalia, 2024) (Fig. 1). Initially, unbiased analysis using the top 2000 variable genes failed to display any distinct trends, likely because of major differences in the total number of cell types found in each of the bulk RNA sequencing datasets. Therefore, we focused the analysis on a previously defined panel of 173 hDRG-specific genes conserved between mice and human (Ray, 2018). This more focused analysis showed different molecular signatures between the cell populations analyzed and that no hiPSC derived sensory neuron population overlapped exactly with primary hDRG, though some datasets were more similar than others.

Since the datasets included contain multiple cell types upon a differentiation continuum, including hiPSCs, neural crest, sensory neurons over differing maturation timeframes, as well as adult human primary DRG; we first explored principal component space to determine which principal components reflected a differentiation trajectory to be able to describe the relative maturation and identity of these populations. We found that while principal component 1 was primarily populated by non-specific housekeeping genes, principal components 2 and 3 provided a rational developmental progression from hPSC to hDRG (Fig. 1). The lower right corners of each plot in Fig. 1 are occupied by hPSCs, indicating the embryologically least mature cell state. Deng et al. 2023 (Deng, 2023) provide valuable embryological context by annotating the differentiation stages in their chronological time course. They describe their 'day 7 population' as 'neural ectoderm,' which are a direct embryological descendant from hPSCs. By 'day 14,' this population differentiates into 'neural crest,' which further matures into the 'nociceptor' population by 'day 56'.

To validate the hypothesis that PC2/PC3 describes an embryological trajectory, we further decomposed each principal component PC2 and PC3 into the genes from which they’re comprised (Fig. 1C and D). Contributors to PC2 and PC3 include known transcription factors corresponding to neurodevelopment, such as SOX2, POU3F1, and SOX10 in PC2; and DLX2, POU4F3, and SOX8 in PC3. Additional genes comprising PC2 and PC3 are related to sensory function, such as nociceptive channels NTRK1 and P2RX3; indicating the analysis to be valid.

Given these landmarks, we can observe in PC2/PC3 that embryological time rotates counterclockwise from the lower right-hand quadrant of the graphs in Fig. 1A and B, with the embryologically most mature populations eventually occupying the bottom-left quadrant of the PC2/PC3 plane. Using this map, we can evaluate which populations are embryologically most mature. The ascending embryological maturation order, therefore, becomes hPSC, Deng days in vitro (DIV) 7, Plumbly DIV 36, Deng DIV 14, Deng DIV 21, Deng DIV 56, Nickolls DIV 36, Anatomic DIV 14, Nickolls DIV 21, Anatomic DIV 21, Anatomic DIV 28, Anatomic DIV 35, Clark DIV 56, Clark DIV 182. As should be obvious from this analysis, the chronology (DIV) doesn’t necessarily predict embryological maturation. Differentiation protocols can be of more advanced embryological age despite a younger chronological age, and vice-versa, reflecting differences between differentiation methods.

To expand upon this point, it appears that differentiation methods reliant upon transcription factor over-expression, the Nickolls et. al 2020 (Nickolls, 2020) and Plumbly et. al 2022 (Plumbly, 2022) datasets, remain embryologically immature and reminiscent of neural crest cells despite an advanced chronological age, which is surprising due to these cells displaying an obvious neuronal phenotype in culture. On the other hand, directed differentiation methods reliant upon embryonic morphogens (Anatomic, Clark et al. 2021 (Clark, 2021), Deng et al. 2023 (Deng, 2023) display the potential to embryologically mature with greater time in vitro. This embryological maturation timescale differs chronologically depending on the protocol, with each protocol having its own maturation timescale. Clark et al. 2021 (Clark, 2021) appears to embryologically mature chronologically faster than Deng et al. 2023 (Deng, 2023), and Anatomic faster than Clark et al. 2021 (Clark, 2021) and Deng et al. 2023 (Deng, 2023).

Notably, no hiPSC-derived population fully overlaps with hDRG in PC space. This could be partially due to the relative heterogeneity of the hDRG, as this dataset was from bulk-sequenced whole DRG, which includes numerous non-sensory neuron cell types such as glia, fibroblasts, and immune cells. However, it likely also represents neuronal differences as ion channels and G protein-coupled receptor (GPCRs) known to be found in sensory neurons were not found in most hiPSC populations.

### Ion channel-, neuropeptide-, and receptor-based comparison of iPSC sensory neurons datasets to native hDRG expression

**Fig. 2:**
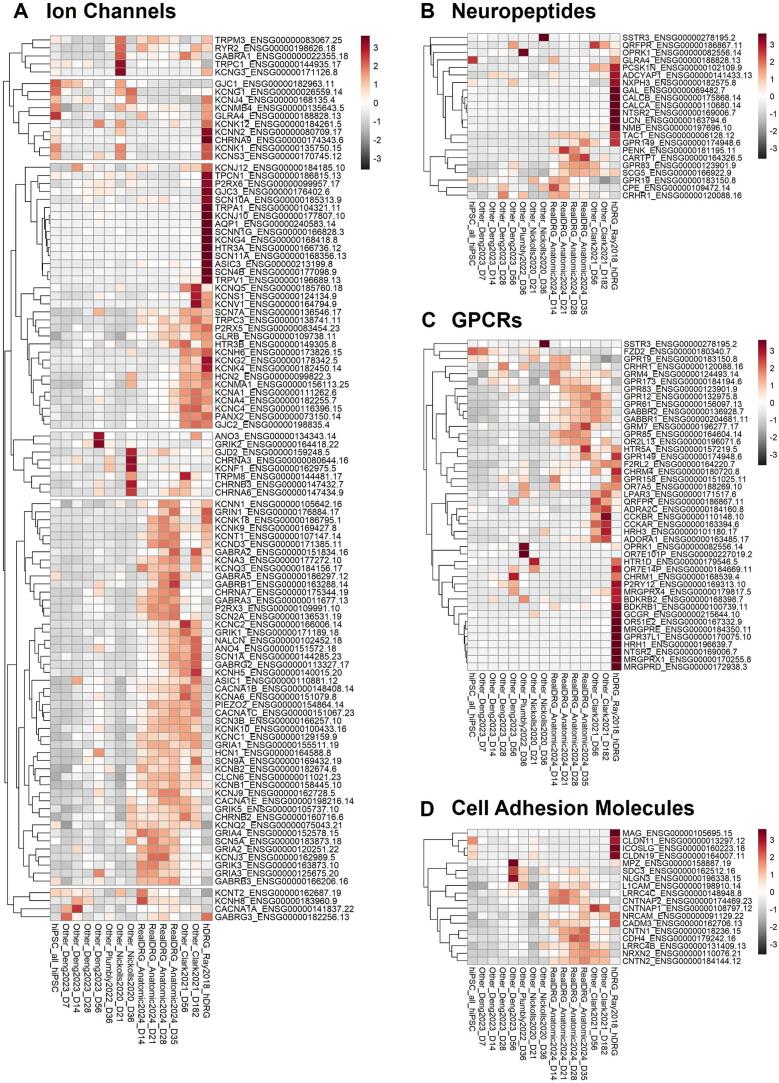
Heatmap of sensory neuron-relevant A) ion channels, B) neuropeptides, C) GPCRs, and D) cell adhesion molecules across primary human DRG, hiPSC-derived sensory neurons, and hiPSCs. Genes are hierarchically clustered based on relative expression, with the dendrogram indicating similarity in expression patterns across datasets.

While the PCA analysis described above gives insight into similarities in transcriptome based on DRG-specific gene expression, pharmacological targets expressed in hDRG including ion channels, neuropeptides, GPCRs, and cell adhesion molecules (CAMs) (Ray, 2018) provided further insight on what differentiates the hiPSC-derived sensory neuron populations (Fig. 2). Hierarchical clustering of curated gene sets reveals relative similarities and differences in expression patterns across hiPSC-derived models and native hDRG. Ion channels play a critical role in DRG neuron physiology. Among these channels are voltage-gated sodium, calcium and potassium channels, transient receptor potential (TRP) channels, mechanosensitive PIEZO channels, and ligand-gated purinergic channels that are all involved in nociceptive signal transduction in DRG neurons. In general, ectoderm-derived hiPSC sensory neurons expressed the most ion channels out of all the hiPSC sensory neuron populations (Fig. 2A). Clark et al. 2021 (Clark, 2021) had also seen expression of a number of ion channels, perhaps due to the longer time of maturation in comparison to other protocols or their differentiation method. The voltage-gated sodium channels SCN9A (Na_V_1.7) and SCN10A (Na_V_1.8) are validated pain targets due to known human genetic mutations which either cause gain-of-function (erythromyalgia) (Cummins et al., 2004, Faber, 2012, Garrison, 2014, Lampert, 2009) or loss-of-function (pain agnosia) (Goldberg, 2007, Cox, 2006, Nilsen, 2009). hiSNs SCN10A expression was highest compared to all protocols though lower than hDRG from Ray et al. 2019. SCN9A expression was comparable in hiSNs and Clark datasets though higher than the hDRG data sets. Nickolls et al 2020 and Clark et al. 2021 (Clark, 2021) protocol had highest expression of SCN11A (Na_V_1.9), which is integral to the fine-tuning of sensory neuron excitability. The full list of voltage-gated sodium channels can be seen in Supplementary Fig. 1A and B. The TRPV1 receptor on C polymodal nociceptors and Aδ mechanoreceptors, and activated by noxious heat and capsaicin leading to burning pain or itch (Willis, 2009), is expressed at the highest level in the Clark et al. 2021 (Clark, 2021) protocol and second highest in the hiSNs population. Notably, no hiPSC-derived sensory neuron expresses TRPV1 to the level of primary hDRG (Supplementary Fig. 1C and D), meaning hDRG far overexpress their hiPSC-derived counterparts given sensory neuron transcripts account for only a fraction of the RNA sequenced in the hDRG samples given their heterogeneity. Interestingly of all the ion channels, Plumbly et. al 2022 (Plumbly, 2022) report a high level of expression of TRPA1, a pain drug discovery target (Koivisto, 2022) involved with the detection of noxious stimuli including teargas, mustard oil and wasabi. TRPA1 is also expressed in non-neuronal cell types involved with inflammation (Yao, 2023) including neural crest-derived melanocytes which could be a contaminant in their cultures (Jia, 2021). A number of other pain targets including voltage-gated potassium channels (KCNQ2/3, KCNQ4, and KCNMA1), the mechanosensitive Piezo2 channel, and ionotropic GABA-A (GABRA2, GABRA3, GABRB3), acid sensing ASIC1 and purinergic receptor channels (P2RX_3_, P2RX_5_) were all highly expressed in hiSNs. In short, a large number of ion channels are expressed in hiSNs that could potentially be used as analgesic drug target screening applications (Jami, 2023, Romero, 2023).

Neuropeptides are often used as markers of subsets of sensory neurons and regulate signaling from DRG neurons to the vasculature and immune cells as well as to dorsal horn neurons in the spinal cord. hiSNs expressed a broader set of neuropeptides compared to other hiPSC-derived sensory neurons, including several higher than hDRG (Fig. 2B). No hiPSC-derived sensory neuron population had expression levels of CALCA and CALCB similar to hDRG, which encode for calcitonin gene-related peptides (CGRP) and are a key migraine pain target for clinical monoclonal antibodies and small molecule receptor inhibitors (Domitrz, 2022, Bigal, 2015, Bigal, 2016, Goadsby, 2017, Ho, 2008, Edvinsson, 2015). In contrast, hiSNs expressed the highest level of TAC1 (tachykinin 1), a gene that encodes the precursor protein of substance P neuropeptide, and also expressed PENK (Proenkephalin), a key precursor to enkephalins which are endogenous opioid peptides that bind to mu-opioid receptors (MORs) and produce analgesia (Henry, 2017, Comb, 1982). These differences suggest that the observed neuropeptide diversity in hiSNs may reflect variations in neuronal subtype representation.

At least 40 members of the approximately 400 non-olfactory G protein coupled receptors (GPCRs) in the human genome are targets for the regulation of pain (Stone and Molliver, 2009, Retamal, 2019). Both hiSNs and the Clark et al. 2021 (Clark, 2021) sensory neurons expressed the most GPCRs related to hDRG (Fig. 2C). hiSNs expressed GRM7 (Glutamate Receptor, Metabotropic 7) and HTR5A (5-Hydroxytryptamine Receptor 5A) which both play a pivotal role in modulating neurotransmitter signaling and sensory processing. OPRK1, or kappa-opioid receptor 1, is a gene that encodes for a protein that is involved in the regulation of pain sensation was also expressed within this population. Clark et al. 2021 (Clark, 2021) highly expressed MRGPRX4 and MRGPRE, genes that encode Mas-related G protein-coupled receptors (Mrgprs) that are involved in the regulation of pain and itch sensations. However, several GPCRs typically expressed in native hDRG were comparatively lower across all hiPSC-derived sensory neurons. As with neuropeptides, this pattern likely reflects differences in neuronal subtype representation or receptor-specific maturation.

Cell adhesion molecules (CAMs) are involved with inflammatory and cell signaling processes and are increasingly viewed as pharmacological targets (Machelska, 2002). hiSNs express the most CAM pharmacological targets including CNTN1, NLGN3, NRCAM, and LRRC4C (Fig. 2D). Deng et. al 2023 (Deng, 2023) and Plumbly et. al 2022 (Plumbly, 2022) have expression of myelin protein zero (MPZ) and myelin-associated glycoprotein (MAG), which are related to myelin formation and maintenance in the nervous system indicating potential Schwann cell contaminants that could originate from the common neural crest progenitor. Together, hiSNs and Clark et al. 2021 (Clark, 2021) sensory neurons expressed the most ion channels, neuropeptides, GPCRs, and cell adhesion molecules (see also Supplementary Fig. 2). While targeted analysis of pain-relevant gene sets highlights areas of alignment with hDRG, global transcriptomic differences remain. These differences likely reflect a combination of incomplete maturation and perhaps the lack of native tissue context, including innervation and circuit-level interactions. As such, transcriptomic similarity should be interpreted as partial and context-dependent rather than comprehensive equivalence; however, the ability to interrogate highly expressed genes of interest as defined targets remains a key strength of the system.

### Single nucleus data with differentiation path framework from day 7–21

**Fig. 3:**
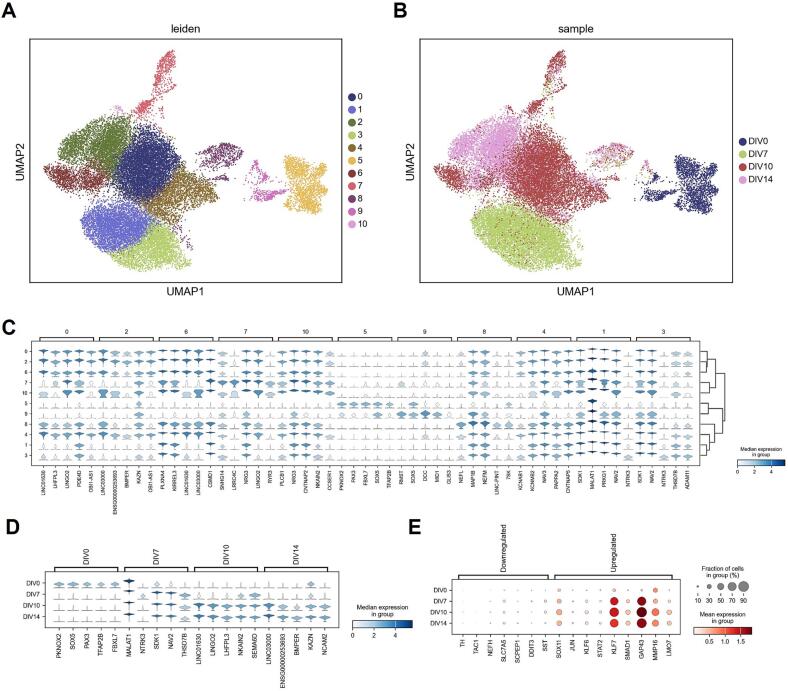
Single nucleus RNA sequencing of 26,733 sensory neurons at DIV7, DIV14, DIV17, and DIV21 post-differentiation reveals distinct transcriptomic signatures across time. A) UMAP plot showing 11 distinct clusters generated by Scanpy. B) UMAP plot showing distribution of individual sample timepoints. C) Stacked violin plot of top five marker genes for each individual Leiden cluster. D) Stacked violin plot of the top five marker genes for each individual timepoint. E) Dotplot showing mature sensory neurons resemble a neuropathic phenotype.

To assess homogeneity of maturing sensory neurons and track expression of key nociceptor markers such as NTRK2, SCN9A, and SCN10A over time we performed single nuclei RNA sequencing on hiSNs cultured to DIV7, DIV14, DIV17, and DIV21 post-differentiation (Fig. 3A). 26,733 nuclei passed best practice quality control, with an average of 2,719 transcripts and 1,610 genes detected per nuclei. Leiden clustering detected 11 distinct clusters across the 4 timepoints. DIV7 and DIV21 cells predominantly populate clusters 5 and 2, respectively; whereas DIV14 and DIV17 are represented across clusters 1 and 3, and 0, 4, 6, and 7, respectively. This seems to suggest an initial divergence from a homogeneous population into heterogeneity, followed by a convergence back into a homogenous population; possibly suggestive of selective events or resolution of transient intermediate fates. This is supported by genes represented in the terminal DIV7 and DIV21 timepoints, whose clusters 5 and 2 respectively are populated by neural crest genes (*PAX3, SOX5, TFAP2B*) followed by neuronal markers (*KCNAB1, KCNMB2, NAV3*) (Fig. 3B). Notably, sample populations show relatively low levels of overlap across DIV, with the exception of Leiden clusters 7 and 8. The top five marker genes for cluster 7, *SNHG14, LRRC4C, NRG3, LINGO2*, and *RYR3*, suggest this cluster may represent cells at an intermediate stage of differentiation towards a nociceptive phenotype. The presence of *RYR3*, a calcium release channel involved in neuronal signaling, and *NRG3*, which is implicated in the development of peripheral nervous system, could indicate these cells are in the process of developing nociceptive functionalities, potentially of the C-fiber subtype. Conversely, the top five marker genes for cluster 8, *NEFL, MAP1B, NEFM, LINK-PINT*, and *7SK*, indicate a possible Aδ-fiber subtype.

Next, we examined the top differentially expressed genes across samples (Fig. 3C). DIV7 cells represented by cluster 5 seem to be in a neural crest or early sensory neuron state. *PKNOX2*, *SOX5*, and *PAX3* are transcription factors that play essential roles in early neural crest development and differentiation. *TFAP2B* is also involved in neural crest cell formation and its expression is a hallmark of early neural crest cells. *FBXL7* has a less well-known role in neural development, but its presence may suggest an active cellular state with ongoing protein turnover and differentiation. At DIV14, the cells appear to be transitioning towards a more mature sensory neuron phenotype. The expression of *NTRK3* (also known as TrkC), a receptor for neurotrophin-3, is indicative of differentiating sensory neurons (Menard, 2018) or low threshold mechanoreceptor neurons. Additionally, *NAV2* and *SDK1* are involved in axon guidance and synapse formation, suggesting ongoing maturation and connectivity of these cells. *MALAT1* is a long non-coding RNA known for its role in regulating gene expression, and its specific role here may be to facilitate this transition phase. By DIV17, the iPSC-derived cells seem to be further maturing into sensory neurons. *LINGO2* is involved in neuronal survival and regeneration, which may indicate ongoing maturation of these neurons. *SEMA6D* is involved in axon guidance, which further suggests maturation and connectivity. *LHFPL3* and *NKAIN2*, while less studied, have been associated with neuronal activity and might indicate functional maturation of these cells. By DIV21, the cells likely represent mature sensory neurons. *BMPER* is involved in the BMP signaling pathway, which is known to play a role in neuronal differentiation and maturation. *NCAM2* plays a role in neuron-neuron adhesion and in synapse formation, indicating that these neurons are likely forming synaptic connections. *KAZN's* role in sensory neurons is less clear, but it is generally involved in cellular homeostasis.

Finally, we examined genes found in specific populations of nociceptors and those implicated in neuropathic pain or nerve injury (Fig. 3D). The decreased expression of *TAC1*, *NEFH*, *SLC7A5*, *SCPEP1*, *DDIT3*, and *SST* suggests a potential lack of representation of neurons that express these markers in hDRG. On the other hand, the upregulation of genes like *SOX11*, *KLF7*, *GAP43*, *MMP16*, and *LMO7* implies an active state of neuronal regeneration or plasticity that is consistent with what is seen with nerve injury in animal models (Fig. 3E).

### Single nucleus data with focus on nociceptor subtypes

**Fig. 4:**
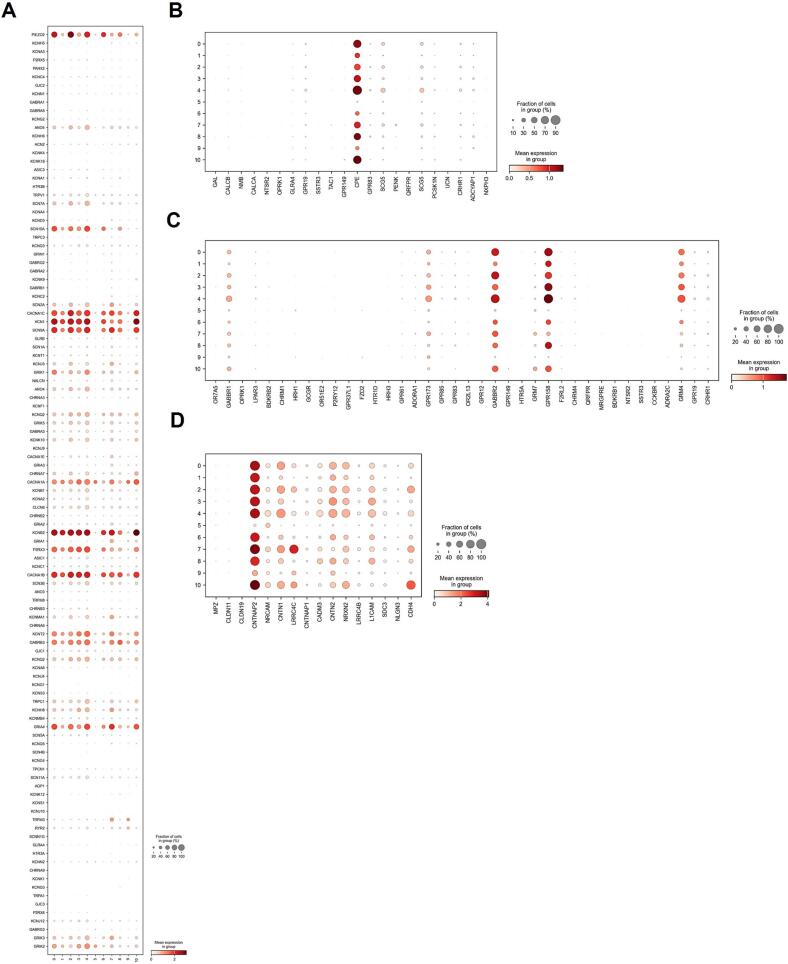
Dotplot analysis showing sensory neuron specific ion channel, neuropeptides, GPCRs, and cell adhesion molecules in single nucleus RNA sequencing Leiden clusters. A) Ion Channels B) Neuropeptides C) GPCRs D) Cell adhesion molecules.

We next chose to look at the previously examined expression of ion channels, neuropeptides, GPCRs, and cell adhesion molecules across identified Leiden clusters to determine the homogeneity of the hiSNs during maturation (Fig. 4). Largely, differences in ion channel expression across Leiden clusters followed the separation of samples across clusters, such as the expression in cluster 5 which is predominantly made up of DIV7 nuclei (Fig. 4A). The ubiquitous expression of *CPE* (Carboxypeptidase E) across all mature clusters from DIV14 onwards suggests a sustained requirement for neuropeptide processing in these differentiated neurons, as *CPE* is instrumental in the biosynthesis of peptide neurotransmitters and hormones (Fig. 4B). Compared to total RNA sequencing, single nuclei sequencing detected abundant levels of relatively few GPCRs, likely due to the lower sequencing depth in the single nucleus experiments (Fig. 4C). Notably, these include *GABBR1*, *GABBR2*, *GPR173*, *GPR158*, and *GRM4, so* the relatively high expression of these GABA receptors suggests these targets may be useful positive controls for GPCR signaling in hiSNs. Further, deorphanizing of *GPR173* and *GRP158* may reveal unique targets for further manipulating cell excitability or explain previously unidentified drivers of signaling in hDRG. Lastly, single nuclei RNA sequencing detected relatively robust levels of cell adhesion molecules (Fig. 4D). Together, these data indicate that across maturation, hiSNs represents a relatively homogenous population with respect to the expression of ion channels and receptors.

### Comparison of single nuclei RNA sequencing cluster 2 to known nociceptive subtype marker genes

**Fig. 5:**
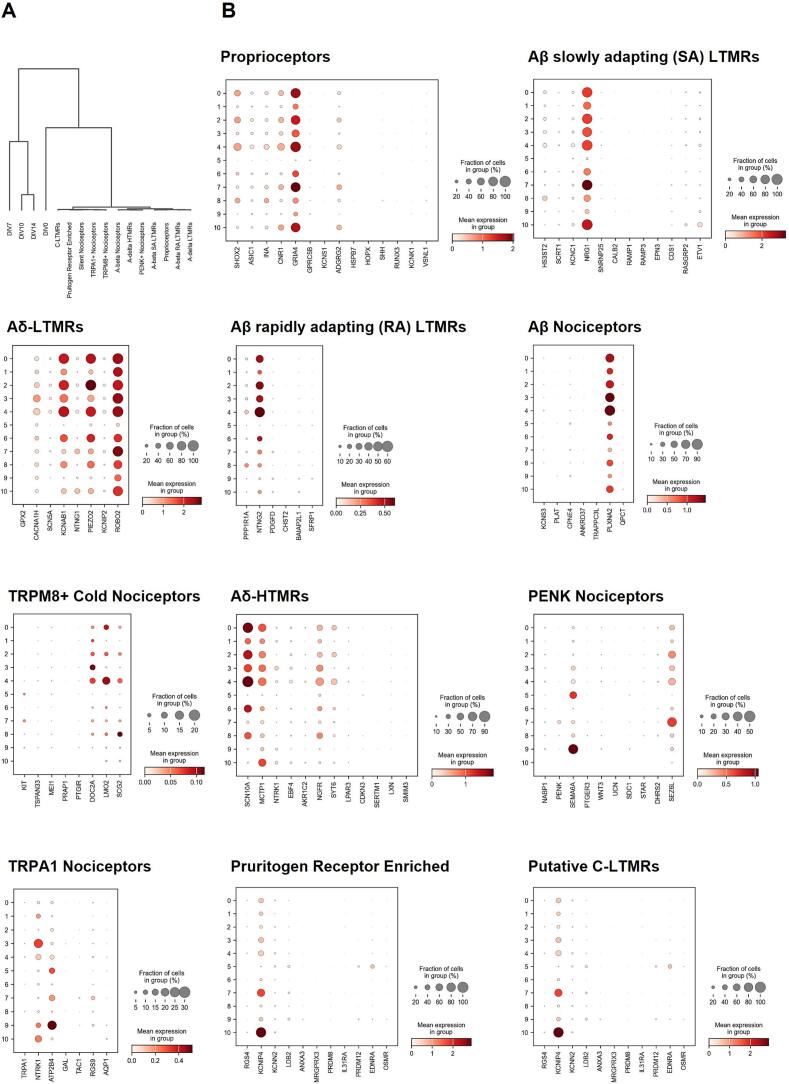
Single nucleus RNA sequencing of hiSNs reveal expression of a range of mechanoreceptor and nociceptor marker genes. A) Dendrogram clustering of hiSNs at DIV7, DIV14, DIV17, DIV21 with previously identified nociceptor subtypes. B) Dotplot expression of nociceptor subtype marker genes across Leiden clusters.

We next sought to understand if terminally differentiated hiSNs represented distinct nociceptor subtypes. First, we integrated a previously generated Visium dataset with proper batch correction using the *bbknn* function in Scanpy and performed a dendrogram clustering of nociceptor subtypes with the samples across time (Fig. 5A). This did not reveal any distinct integration of hiSNs samples with nociceptor subtypes. Next, we examined expression of key differentially expressed genes from nociceptor subtype clusters as previously performed with total RNA sequencing. Most notably, hiSNs express a range of Aδ-LTMR genes (KCNAB1, PIEZO2, ROBO2), Aδ-HTMR genes (SCN10A, MCTP1, NGFR), and select genes found in various nociceptor populations (PLXNA2, NTRK1, ATP2B4, KCNIP4) (Fig. 5B). Given that the monolithic population found in cluster 2, representing the most chronologically aged state in our dataset, appears to encompass multiple discrete populations found in the adult, we expect these neurons have yet to resolve their fate at this time point and possibly require additional maturation to determine their eventual subtypes. Regardless, it could be said that these immature neurons possess a dual identity reminiscent of both mechanoreceptors and nociceptors. Sequencing of later timepoints would inform on whether a fate decision is eventually made to be either nociceptive, mechanoreceptive, or a mixed population following a divergence. The absence of clear alignment with defined nociceptor subtypes suggests that these cells may represent an intermediate or unresolved state, rather than fully specified adult subpopulations. Additional maturation or environmental cues may be required to drive further subtype specification.

### Functional characterization of hiSNs using automated patch clamp

**Fig. 6:**
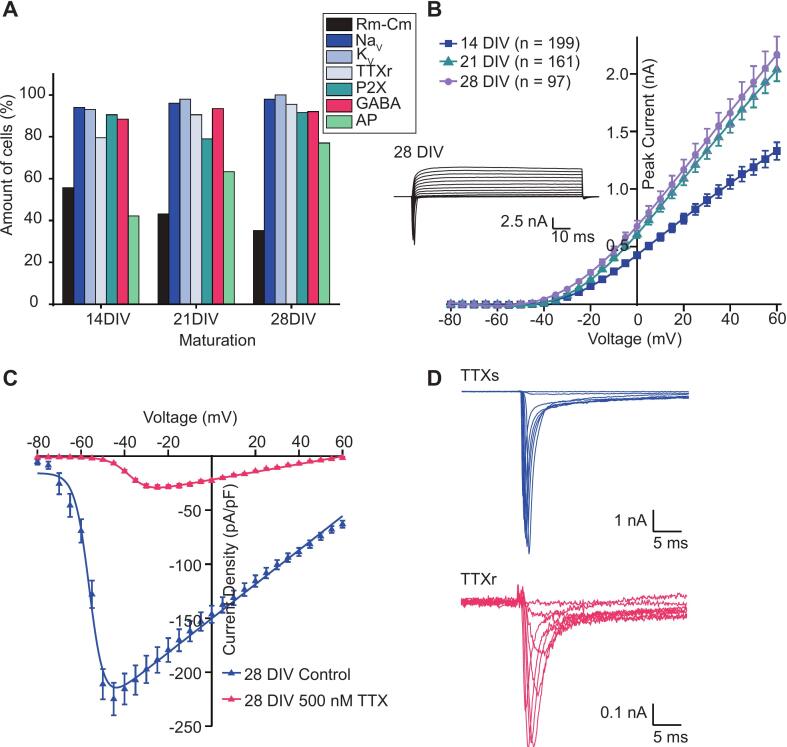
Voltage-gated currents recorded from hiSNs sensory neurons using APC. A. Summary of experimental success rates for cell capture (Rm > 300 MΩ and Cslow > 2 pF), cells with K_V_, Na_V_ currents, P2X, GABA-A ligand-gated ionotropic receptor responses, and action potentials (AP). There appear to be some changes over time. B. K_V_ current–voltage plot at 14, 21 DIV and 28 DIV recorded on the SyncroPatch 384 with corresponding traces from an exemplar cell at 28 DIV. C. NaV current density at DIV 28 in the absence (dark blue) and presence (red) of TTX. D. Example traces of TTXs (top) and TTXr (bottom) from an exemplar cell. The differences in decay kinetics are clearly visible. (For interpretation of the references to colour in this figure legend, the reader is referred to the web version of this article.)

Recent work has demonstrated the feasibility of applying high throughput automated patch clamp (APC) techniques to study voltage-gated sodium (Na_v_) channels in native rodent DRG neurons (Ghovanloo, 2023) and hiSNs (Kalia, 2023). In order to unlock the full potential of this technique for translatable, high throughput pain drug discovery, we looked to improve previously published success rates and expand the range of ion channel targets that can be functionally detected in hiSNs over a course of maturation using three APC systems: the low throughput Port-a-Patch, the medium throughput Patchliner, and the high throughput SyncroPatch 384. Using an optimized cell dissociation protocol we were able to obtain success rates ranging between 35–60 % for various experimental parameters (see Methods) in cells matured over 14–28 days in vitro (DIV) (Fig. 6A). Inward Na_V_ (including TTX-sensitive and TTX-resistant components), outward K_v_ currents and functional ligand-gated P2X and GABA-A receptor-mediated responses were seen in 80–100 % of cells, while cells showing action potential firing increased from 40 to 80 % over 3 weeks (Fig. 6A). Seal quality was excellent (Quality control (QC) acceptance: R_Seal_ > 300 MΩ; Table 1) and high throughput sampling was feasible from over 100 cells per run on the SyncroPatch 384 device, enabling comprehensive statistical analysis of hiSNs in less than an hour.

**Table 1:** Electrophysiological and functional parameters for hiSNs at different maturation times (14, 21 or 28 DIV) recorded on the SyncroPatch 384. Current density for Na_V_ was recorded at 0 mV; TTXr was recorded at 10 mV; K_V_ was recorded at 40 mV; P2X-mediated currents were recorded at a holding potential of −80 mV in response to 20 µM adenosine triphosphate (ATP) for 1 s; and GABA-A currents were recorded at a holding potential of −80 mV in response to 100 µM GABA for 1 s. Number of cells for each parameter is given in parentheses, values are given as mean ± S.E.M.

Parameter	14 DIV	21 DIV	28 DIV
Seal Resistance (GΩ)	1.1 ± 0.07 (214)	0.96 ± 0.1 (164)	1.0 ± 0.1 (97)
Cell Capacitance (pF)	8.0 ± 0.5 (220)	12.0 ± 0.6 (168)	16.3 ± 2.9 (103)
Na_V_ current density (pA/pF)	314 ± 15 (202)	314 ± 16 (160)	235 ± 20 (235)
TTX_r_ current density (pA/pF)	30.2 ± 3.4 (155)	33.6 ± 1.7 (143)	28.4 ± 1.7 (89)
K_V_ current density (pA/pF)	144 ± 4.8 (202)	159 ± 5.6 (160)	151 ± 6.9 (97)
P2X current density (pA/pF)	83.8 ± 5.4 (94)	49.2 ± 2.9 (97)	47.4 ± 3.4 (65)
GABA-A current density (pA/pF)	130 ± 9 (136)	121 ± 12 (100)	207 ± 49 (13)

### Voltage-gated ion channels recorded from hiSNs using automated patch clamp

DRG sensory neurons express a wide range of voltage-gated inward Na_V_ and Ca_V_ and outward K_V_ currents that control pain transmission, which can be accurately measured in voltage clamp mode using APC (Fig. 6B-D). TTX-sensitive Na_V_1.7 (SCN9A), and TTX-resistant Na_V_1.8 (SCN10A) and Na_V_1.9 (SCN11A) channels are of particular interest for analgesia, as mutations in these targets are associated with various pain states in human patients (Bennett, 2019). Further, Na_V_1.5 (SCN5A) expression in DRG has been documented and a recent publication posits a role for this channel in mechanical hypersensitivity in neuropathy (Gomes-Aragão, 2026, Zhang, 2026, Renganathan et al., 2002). hiSNs sensory neurons express SCN9A and SCN10A RNA, with lower levels of SCN11A, and voltage clamp recordings on the SyncroPatch 384, Patchliner and Port-a-Patch show large TTX-sensitive currents activating at negative potentials, and a significant (∼10–20 %) component of TTX-resistant inward current that activates at more positive potentials, characteristic of Na_V_1.8 channels and matching previously published results (Kalia, 2024) (Fig. 6C and D). The kinetics of TTX-resistant currents in hiSNs, and their relative contribution to macroscopic I_Na_, are similar to those recorded from adult human DRG (Zhang, 2017) but differ from that in rat DRG neurons, illustrating another advantage of recording from native cells on APC to elucidate key species-specific differences. While the functional expression of multiple ion channel classes supports the utility of this system for pharmacological interrogation, these measurements represent only a subset of neuronal function and do not capture the full physiological complexity of sensory neurons in vivo.

### Ligand-gated ion channels recorded from hiSNs neurons

**Fig. 7:**
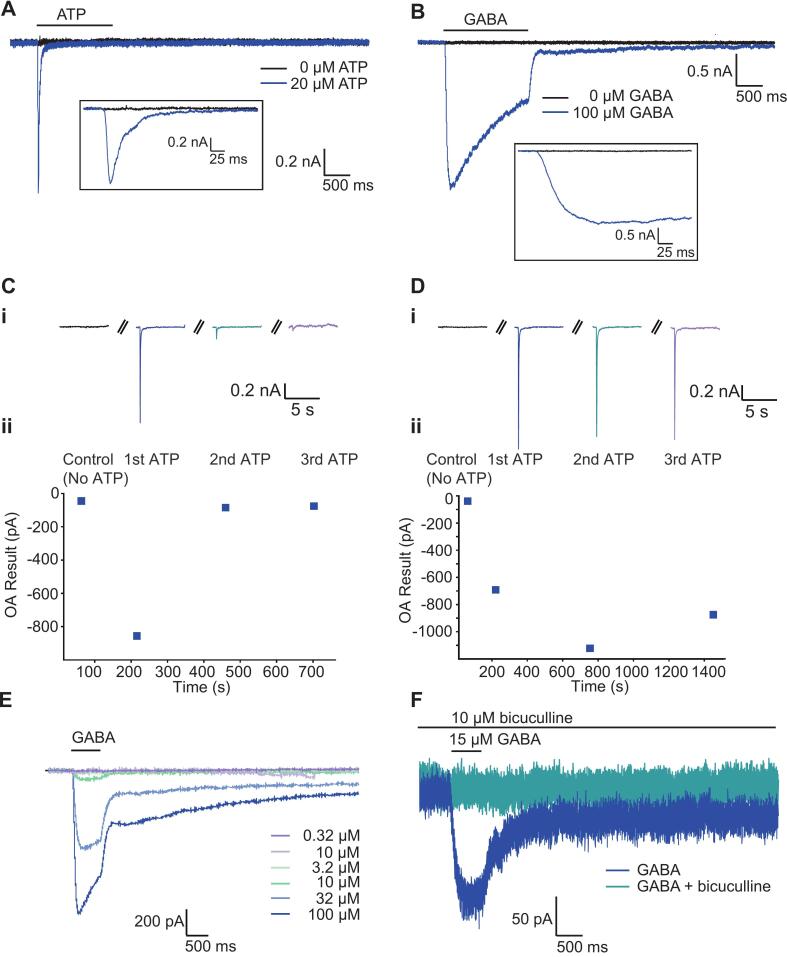
Ligand-gated ionotropic receptor currents. A) P2X receptor currents activated using 20 µM ATP on the SyncroPatch 384. Rapid activation and desensitization of ATP-evoked currents; inset at expanded time scale. B) GABA-A receptors activated by fast application of 100 µM GABA on the SyncroPatch 384. Inset shows time course of activation on an expanded time scale. C) Desensitization of P2X currents evoked every 4 min. This can be reduced to give consistent repeated responses in the same cell if ATP applications are made every 10 min (D). Note: all recordings of P2X were performed in the presence of hexokinase to prevent ATP degradation. (E) Currents activated by GABA in a dose-dependent manner and (F) inhibited by the competitive GABA-A antagonist bicuculline (10 µM).

To further demonstrate translational utility of hiPSC-derived nociceptors, we investigated the pharmacological responses of several ligand gated ionotropic targets that were highly expressed in hiSNs neurons and found in native rodent and human DRG sensory neurons. Due to the ability to rapidly apply and wash-off small liquid volumes with the robotic liquid handlers on the Patchliner and SyncroPatch 384 devices, we focus here on two validated DRG sensory neuron targets, P2X and GABA-A ionotropic receptor channels (Fig. 7).

P2X_3_ receptor antagonists are promising drugs for the treatment of refractory chronic cough (Richards, 2019). There are major differences in gene expression of P2X receptors between rodent and human DRG (Shiers et al., 2020). On the SyncroPatch 384 and Patchliner, we observed the expected rapid activation and inactivation of inward currents upon application of ATP which is consistent with P2X_3_-mediated currents (Khakh, 2001, Spinaci, 2021) (Fig. 7A). In addition, we observed profound desensitization to repeated stimuli with ATP (Fig. 7C), but this effect could be effectively removed by extending the time between stimuli (Fig. 7D), resulting in stable responses to repeated ligand application suitable for drug screening. We used ATP to activate P2X receptors to activate a broad range of channels and although the fast kinetics of the channel indicate P2X_3_ as the underlying subtype, this would need to be confirmed using pharmacology (activation by α,β-methylene ATP and/or inhibition by selective antagonists e.g. A-317491 (Spinaci, 2021, Jarvis, 2002) in a follow-up study.

GABA-A ionotropic receptors are broadly expressed throughout the nervous system and have been associated with a wide variety of psychiatric and neurodevelopmental disorders including neuropathic pain (Li, 2019). Although DRG neurons lack intra-ganglionic synapses, GABA is a local neurotransmitter in this system and GABA-A Cl^-^ channels are expressed in adult rat (Du, 2017) and human DRG neurons (Tavares-Ferreira, 2022) and their activation opens inward Cl^-^ currents that depolarize embryonic and hyperpolarize postnatal DRG neurons. GABA-A receptors can also be targeted by a wide variety of pharmacological compounds including benzodiazepines and general anesthetics. hiSNs express GABA-related genes GABRA2, GABRA3, GABRB3, GABBR1, GABBR2, and we successfully recorded currents in response to application of 100 µM GABA that display the expected activation and inactivation kinetics (Fig. 7B). As with the P2X recordings, GABA-A responses were detected in ∼90 % of hiSNs, with current densities in the range of 125 – 200 pA/pF for GABA at 100 µM (Fig. 6, Fig. 7). The response to GABA was concentration-dependent (Fig. 7E) and blocked by the antagonist bicuculline (Fig. 7F) confirming that the responses are mediated by GABA-A receptors, although the subunit combination was not investigated.

### Mechano-activated currents in hiSNs are likely mediated by Piezo1 and Piezo2

**Fig. 8:**
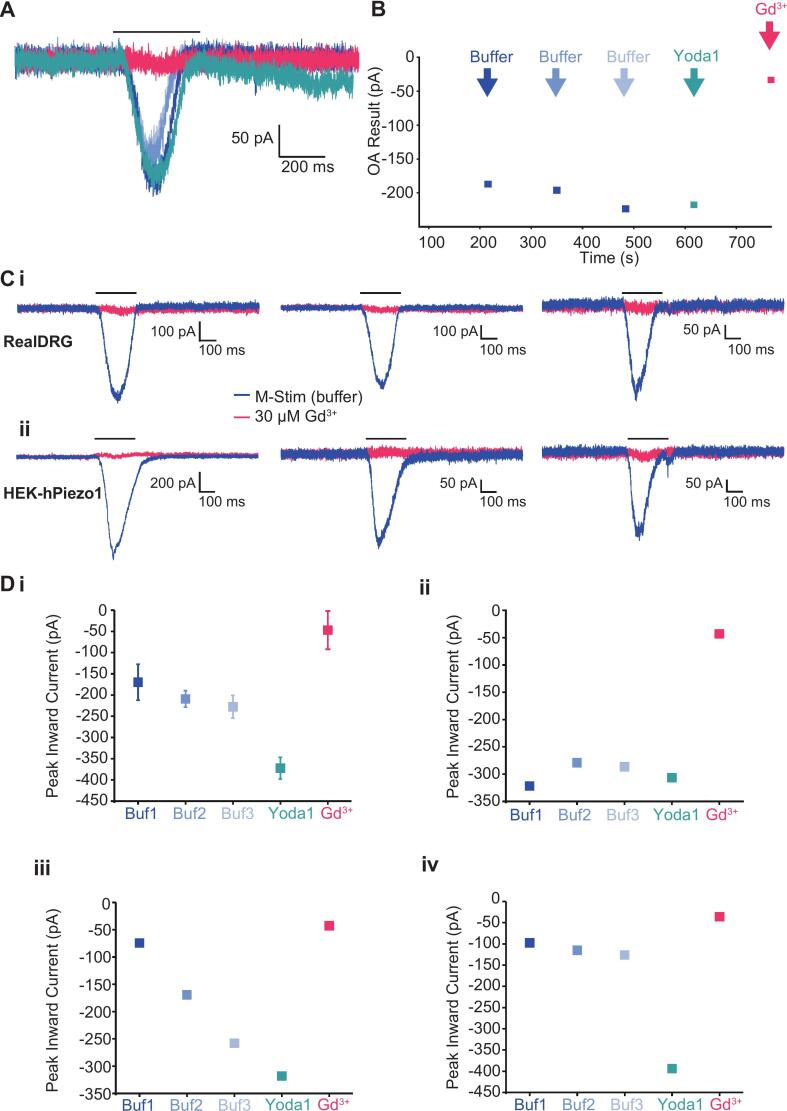
Mechano-gated ionic currents in hiSNs. A) Current traces recorded from an exemplar hiSNs on the SyncroPatch 384 shown overlaid where blue traces are buffer-only, green trace is with Yoda1 and red trace is with GdCl_3_. B) Timecourse of the experiment indicating activation using mechanical stimulation (M−Stim) where buffer solution was pipetting 110 µL/s at −80 mV. This was repeated 3 times followed by a 4th application containing Yoda1 at 10 µM and a final application containing GdCl_3_ at 30 µM. Ci) Sample current traces from Real DRG neurons and comparison with ii. HEK cells overexpressing hPiezo1 in response to M−Stim protocol. Blue traces are currents in response to M−Stim with buffer only and red traces are currents in response to M−Stim in the presence of 30 µM GdCl_3_. D) Plot of peak inward current over time during M−Stim protocol. Di represents average ± SEM from all cells with current > 50 pA in response to buffer alone. ii – iv are representative examples of individual neurons highlighting various response profiles: ii shows stable responses with no Yoda1 effect, iii shows current sensitization over time with no clear Yoda1 effect and iv shows stable responses to buffer followed by Yoda1 potentiation. All currents were blocked by GdCl_3_. (For interpretation of the references to colour in this figure legend, the reader is referred to the web version of this article.)

Sensory neurons are highly sensitive to mechanical forces and are important for processing touch, proprioception and mechanical pain. Piezo ion channels are non-selective cation channels that transduce mechanical stimuli into electrical signals (Ranade et al., 2015). These channels open rapidly in response to membrane stretch and undergo rapid inactivation during continued application of the mechanical stimulus (Zheng et al., 2019). Piezo2 channels are thought to be the primary transducer of mechanical touch sensation in sensory neurons, and expression of Piezo2 mRNA and functional channels have been demonstrated in hiSNs (Romero, 2023, Gabrielle, et al., 2024). While Piezo1 channels have been mostly implicated in mechano-sensation in non-neuronal cell types such as red blood cells and mRNA expression is lower in hiSNs, there have been reports of Piezo1 expression and activity in DRG neurons (Roh, 2020, Shin, 2021). Recently a new technique, M−Stim, for high throughput APC measurements of Piezo channel activity was developed in which fluid flow shear stress is applied acutely to cells under patch clamp, which has been applied to Piezo1 channels expressed in HEK cells (Murciano, 2023). We sought to extend the M−Stim technique to evaluate the presence of mechanically gated channels in hiSNs. Briefly, the automated liquid handling robot was programmed to deliver brief pulses of shear stress (20 µL at 100 µL/s) repeatedly. The standard protocol involves 3 rounds of stimulation with external buffer alone, a fourth application of buffer plus the Piezo1- selective potentiator, Yoda1, and a fifth application of buffer with GdCl_3_ as a non-selective blocker of Piezo channels (Fig. 8A and B). Remarkably, 65 % of all successfully patched cells survived all 5 rounds of M−Stim shear stress representing an overall success rate of 37 %. Among these cells that survived the entire M−Stim testing paradigm, 31 % had rapidly activating and inactivating inward currents of 50 pA or greater that were blocked by Gd^3+^. Fig. 8C displays representative traces from 3 hiSNs along with 3 Piezo1- expressing HEK cells to the same M−stim protocol, highlighting the similarities in activation and inactivation kinetics between native sensory neuron and heterologously expressed mechanosensitive currents. A larger proportion of hiSNs (54 %) responded to M−Stim in the presence of Yoda-1 which suggests the presence of functional Piezo1 channels in these cells. On average, the current amplitude in the presence of 10 µM Yoda1 was ∼80 % larger than in its absence (Fig. 8Di). In contrast, M−Stim measurements of Piezo1 channels heterologously expressed in HEK cells showed 400–800 % larger currents in the presence of Yoda1, indicating that Piezo1 channels are responsible for only a fraction of the total mechanosensitive current in hiSNs, consistent with the possibility that Piezo2 channels are the major contributor to mechano-gated ionic currents in hiSNs which is supported by the high levels of Piezo2 expression. Lastly, analysis of individual hiSNs responses to M−Stim and Yoda1 revealed a variety of responses, ranging from stable current with no modulation by Yoda1 (Fig. 8Dii) or potentiation over time with no apparent modulation by Yoda1 (Fig. 8Diii), to some cells which showed large potentiation by Yoda1 (Fig. 8Div).

### Action potential firing in hiSNs neurons

**Fig. 9:**
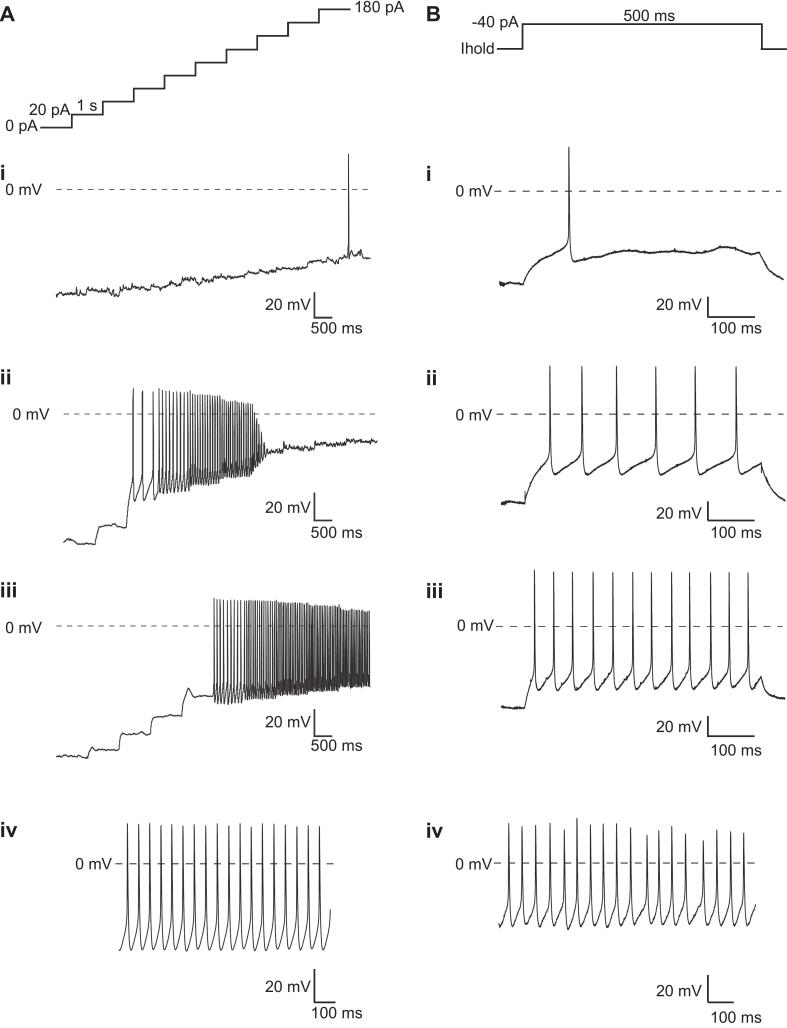
Elicited and spontaneous action potentials in hiSNs. A. Staircase protocol used to elicit action potentials on the SyncroPatch 384. Injected current was increased by 20 pA in 1 s steps to find the threshold for eliciting action potentials in each cell. Different action potential profiles for individual cells recorded on the SyncroPatch 384 at 21 or 28 DIV, ranging from a single action potential at a given current (i), bursts of action potential firing (ii – iii). iv. Spontaneous action potentials were observed in a small subset of hiSNs neurons when no current was injected on the SyncroPatch 384 (RMP approx. −52 mV). B. Single step protocol used to elicit action potentials on the Patchliner. Different action potential profiles for individual cells recorded on the Patchliner at 21 or 28 DIV, ranging from a single action potential at a given current (i), bursts of action potential firing (ii – iii). iv. Spontaneous action potentials were observed in a small subset of hiSNs neurons when no current was injected on the Patchliner (RMP approx. −45 mV).

The ability to examine human neurons using current clamp to measure action potential firing and assess changes in excitability complements voltage clamp analysis of ionic currents measured under similar conditions on each APC platform. Following a detailed characterization of voltage-gated, ligand-gated and mechano-activated currents in voltage clamp mode, we investigated the ability of hiSNs to fire action potentials in current clamp mode on Patchliner and SyncroPatch 384 APC devices. In a small number of hiSNs, spontaneous action potentials were recorded (Fig. 9Aiv and Biv), although these were only observed in 3.3 % or 3.5 % of cells at 21 and 28 DIV, respectively as expected from native sensory neurons. In order to elicit action potentials, cells were held with holding current to maintain a resting membrane potential of −90 mV and then a staircase current protocol shown in Fig. 9A or a step protocol shown in Fig. 9B was used to investigate the threshold at which action potentials were elicited in each cell. Fig. 9A and B show several examples of different firing profiles in hiSNs on the SyncroPatch 384 and Patchliner, suggestive of possible functional heterogeneity within the population, potentially related to differences in cell identity, ionic current expression or maturity. Complex ramp and step protocols can be utilized to assess changes in membrane excitability which are known to be involved in pathological or pain signaling. This enables detailed categorization of single sensory neurons based on their action potential shape, as well as their ability to respond to patho-physiological stimuli and pharmacological agents. Action potential firing in hiSNs was abolished upon application of 500 nM TTX as expected after block of NaV channels (Supplementary Fig. 3).

## Discussion

This comparative study provides molecular and functional insights into hiPSC-derived sensory neurons as a human-relevant model for pain research. No published hiPSC-derived sensory neuron protocol completely recapitulates primary hDRG tissue on a bulk RNA or protein expression level, perhaps unsurprising as hDRG tissues also contain a number of other cell types like satellite glia, Schwann cells, and immune cells with diverse transcriptomes. The goal of this study is therefore not to demonstrate equivalence, but to define where these models align with, and diverge from, human DRG, and how they may be appropriately applied in translational research contexts.

The presence of non-sensory cells in hDRG are expected to dilute the total amount of RNA contributed by sensory neurons, highlighting the extremely high levels of nociceptive transcripts present in native hDRG sensory neurons in comparison to any hiPSC-derived sensory neuron. To benchmark hiPSC-derived sensory neurons against native hDRG, therefore, it would be expected the expression level for any transcript found in a pure population of hiPSC-derived sensory neurons would likely need to exceed the expression found in hDRG for the two populations to approach parity. Alternatively, bulk-sequenced hiPSC-derived sensory neurons could be benchmarked against pseudo-bulked single-cell transcriptional analyses of native hDRG to understand relative expression levels, and therefore parity. Considering these factors, the data from hiSNs appears to capture a subset of transcriptional features associated with human DRG, with alignment dependent on the gene sets and comparisons used. Furthermore, numerous nociceptive transcripts found in hiSNs have been shown to be functional by high throughput APC, demonstrating an expression-function relationship. Given the scalability, easy availability and relatively low cost of hPSC-derived systems compared to accessing hDRG samples, hiSNs provide a scalable and target specific resource for drug discovery for analgesia and other human disease-related targets such as those that appear in this publication.

With many hiPSC-derived cell types, time in culture is often used as a solution toward embryological maturation. The present findings suggest this to be true assuming a directed differentiation methodology is used, such as with the Clark et al. 2021 (Clark, 2021); Deng et al. 2023 (Deng, 2023), and Anatomic cell protocols. However, maturation timelines can be accelerated when using directed differentiation depending on the environment to which the cells are exposed. The Anatomic protocol appears to accelerate embryological progression into an immature sensory neuron, which then can be accelerated toward maturation through time in comparison to the other directed differentiation protocols.

Future refinements to maturation post-differentiation will need to be made to further increase nociceptive transcripts, with the idea that the transcription level needs to be increased to parity with hDRG to demonstrate functionality. Refinements could come in the form of additional soluble factors present in the media that more closely mimics the in vivo environment, or the addition of supportive cell types in co-culture found in the peripheral sensory nervous system, such as satellite glia and Schwann cells. Recent work demonstrates that direct contact with satellite glia enhances differentiation, electrophysiological maturity, and transcriptional programs in hiPSC-derived sensory neurons, while also altering functional responses to injury-relevant stimuli, including increased susceptibility to paclitaxel-induced axonal degeneration, highlighting the role of the cellular microenvironment in modulating pharmacological and disease-relevant phenotypes (LeBlang, 2025). More broadly, injury and disease contexts underscore the importance of neuron-microenvironment interactions, where nerve injury-associated signaling can drive transcriptional and functional remodeling (LeBlang, 2025, Baruch, 2025). It is possible the innervation targets of sensory neurons also play a role in their maturation, so the inclusion of peripheral targets such as keratinocytes or central targets such as dorsal horn neurons will need to be studied. Finally, the unique anatomy of sensory neurons may need to be recapitulated, possibly with multi-compartment microfluidic chips, to further increase anatomic, structural and synaptic fidelity to their in vivo counterparts (Kimourtzis and Raouf, 2024, Mutschler, 2023, Vysokov et al., 2019). These new lines of inquiry are beginning to be explored, and provide great potential to improve present culture systems. It is becoming clear, however, that strategies involving exogenously over-expressed identity transcription factors may have inherent limitation in achieving improved culture performance, particularly when compared to existing methods using directed differentiation. The present work finds that these former iPSC sensory neuron populations, even after advanced chronological aging, appear to be more transcriptionally aligned with the earlier developmental neural crest stage, without clear progression toward adult human DRG. Given transcription generally precedes translation and function, this incomplete transcriptional alignment may contribute to their limited functional relevance and utility in pain drug discovery. To summarize the two methodologies used to differentiate hiPSCs as it relates to maturation, for sensory neurons at least, it appears the embryological “journey” encoded by morphogens is more important than the “destination” transcription factors which need to be overexpressed.

While modulating expression levels in a model system can be useful for predicting therapeutic efficacy in modalities such as antisense oligonucleotide therapy, scientists require a functional model that translates physiologically and pharmacologically. Utilizing several high throughput APC devices, we demonstrate that a number of pain-related targets such as voltage-gated sodium and potassium channels, ligand-gated GABA-A and P2X receptors, and now mechanosensitive Piezo channels, can be functionally characterized and assayed with high success rates and reproducible functional readouts. For example, the functional expression of TTX-sensitive (Na_V_1.7 channel) and TTX-R (Na_V_1.5 or Na_V_1.8 channels) currents, and good correlation with expression of their respective SCN9A, SCN5A and SCN10A genes, supports the use of hiSNs as a translationally relevant system for pain ion channel focused drug discovery. Importantly, APC provides a single-cell, high-resolution electrophysiological readout that complements analgesic screening in hiPSC-derived sensory neurons using multielectrode arrays (MEA) (Fofie, 2025). While MEA captures population-level activity through time and pharmacological responses, APC enables precise dissection of ion channel function at the single-cell level, together providing a more comprehensive framework for evaluating candidate therapeutics. Furthermore, the successful combination of iPSC sensory neurons and APC allows for efficient pharmacological testing of key sensory neuron ion channels, and may be a technique that can be optimized to interrogate other novel pain targets of interest such as TRP channels or intracellular Ryanodine receptors (Haji-Ghassemi, 2023).

In conclusion, integrating transcriptomics with functional electrophysiology offers a robust approach to explore the molecular and cellular mechanisms underlying pain and to identify novel analgesic targets. While these systems do not fully recapitulate native hDRG, they enable targeted interrogation of defined molecular and functional endpoints in a scalable and experimentally accessible format. Sex-dependent differences in sensory neuron biology are increasingly recognized, particularly in pain mechanisms and pharmacological responses (Chemin, 2026, Bartley and Fillingim, 2013), and are not captured here due to the use of a single donor line; however, the rapid differentiation timeline and scalability of this platform may facilitate future studies incorporating multiple donors and stratified analyses. Continued refinement of maturation strategies, microenvironmental context, and multi-modal characterization will be important for improving the predictive and translational utility of these hiPSC-derived sensory neuron models.

## Materials and methods

### Differentiation of hiPSC sensory neurons

As previously described (Kalia, 2023), the female hiPSC line (ANAT001) was cultured under feeder-free chemically defined conditions using TeSR-E8 medium and vitronectin as a coating substrate. Cells were single cell passaged onto a defined matrix and cultures were fed daily with a scaled-up version of Anatomic’s Senso-DM to produce immature sensory neurons by day 7 post-induction. Day 7 cultures were dissociated and cryopreserved. Lot-specific metrics were recorded including yield, cell number per vial, viability, post-thaw recovery, post-thaw viability, post-thaw morphology, purity, and sterility. Criteria used to determine lots passing quality control included neuronal purity > 95 %, verified cell number per vial, post-thaw viability > 70 %, and sterility. Additional detail related to differentiation, media compositions, materials used, and bioprocessing steps are proprietary information of Anatomic.

### Bulk RNAseq analysis

Three lots of hiSNs manufactured from the same hiPSC line were thawed in Senso-MM (Anatomic Incorporated, #1030) at 100,000 neurons/cm^2^ in T25 format. Total RNA was collected at weekly timepoints for each line through four weeks. Pellets were formed by swirling cultures to release the cultures as a sheet before collection and centrifugation at 300g for 5 min. Cells were lysed and RNA extracted according to the RNA isolation kit instructions (Qiagen, #74104). After all samples had been collected, sequencing libraries were generated using Illumina TruSeq Stranded mRNA Library Prep Kit. Equimolar quantities from each library were sequenced on a NextSeq 550 High Output Kit v2.5 (75 cycles) at approximately 30 million single-end reads per sample. Publicly available datasets were retrieved from the SRA using the SRA-Toolkit (https://hpc.nih.gov/apps/sratoolkit.html). When available, paired end-reads were retrieved. For samples from Ray *et al.*, FASTQ files were uploaded to an S3 bucket and verified with md5sum hashes before processing. All samples were processed using the nf-core RNA-Seq pipeline (v 3.11.2) (Ewels, 2020, Patel, 2024). Nextflow (v22.10.6) (Di Tommaso, 2017) using the ‘--docker‘ profile to execute the workflow. Release 109 of the Human GRCh38 primary assembly was retrieved from ftp.ensembl.org. Release 43 of Gencode annotations for the Human primary assembly were retrieved from gencodegenes.org. Reads were aligned with STAR (v2.7.10a) and quantified using RSEM (v.1.3.1). In total, over 42 billion reads passed QC, or the equivalent of over 100 NextSeq lanes. For each sample, reads passing quality filters were used to generate transcript annotations using StringTie (v2.2.1). The Gencode annotation gtf was used to guide transcript assembly. Next, all novel transcript assemblies were merged and compared to the Gencode annotation using gffcompare (v0.12.6). The transcript map (.tmap) file was processed in R and class ‘s’ transcript annotations were discarded. RSEM quantifications of both gene and transcript level counts were loaded using the tximeta (v1.16.1) package with R 4.2.2. Transcripts with minimal expression (< 5 counts across all samples) were discarded. The function DESeqDataSetFromTximport was used to import quantifications and create a DESeq2 (v1.38.3) object for differential transcript and gene expression analysis. Gene sets used for transcriptomic comparisons were curated based on previously reported sensory neuron-relevant gene families, including ion channels, GPCRs, and CAMs (Ray, 2018). Expression values were extracted across datasets and visualized using hierarchical clustering to assess relative expression patterns across hiPSC-derived sensory neuron models and native human DRG samples. Primary human DRG datasets used for comparison were derived from adult post-mortem donors (e.g., Ray et al., 2018), with reported ages spanning early adulthood to older individuals where available.

### Single cell RNAseq analysis

Cells were cultured according to methods to DIV0, DIV7, DIV10, and DIV14. Adhered sensory neurons were washed from cell culture plates with PBS and centrifuged at 500g for 5 min at 4°C to form a pellet. Following pellet aggregation, cells were lyzed and nuclei were isolated according to manufacture protocol for the Nuclei Isolation Kit: Nuclei EZ Prep (Sigma-Aldrich). Nuclei were then passed through a 40 µm strainer to remove cellular debris and fixed according to manufacturer protocol within the Parse Biosciences fixation kit. Fixed nuclei from each collection point were stored at −80°C in a Mr. Frosty Freezing Container (Thermo Scientific). After all samples had been collected, sequencing libraries were generated using the Evercode Whole Transcriptome 100 k v1 Kit (Parse Biosciences). Each sample used 12 of the available 48 wells of the library preparation kit, and individual nuclei were distributed evenly among wells. The libraries were sequenced on a NextSeq 550 High Output Kit v2.5 (300 Cycles) with a single 6 bp index. Prior to sequencing, a 5 % PhiX spike-in was added according to manufacturer suggestion. Demultiplexing of sequencing libraries was performed using the Parse Biosciences Pipeline (v1.0.5p) to generate count matrices for individual samples. All downstream analysis including quality control, dimensional reduction, leiden clustering, and differential gene expression was performed within the Scanpy package (v1.9.2).

### Automated patch clamp recordings

Cells were thawed and plated into T75 flasks coated with iMatrix 511-SILK at a seeding density of 40,000 cells per cm2 and matured in Senso-MM. Cells were harvested using the Worthington Papain Kit (Worthington Biochemical Corp., Lakewood, NJ, USA) with slight modifications to achieve gentle dissociation of the neurons. Briefly, the neurons were incubated with 5 mL/T-75 flask of the papain/DNAase solution for 75 min at 37C followed by gentle trituration until free floating individual soma were observed. This suspension was carefully layered onto 6.5 mL of ovomucoid inhibitor and centrifuged for 3.75 min at ∼190Xg. The cell pellet was resuspended with standard physiological external solution at densities between 50,000 – 80,000 cells/mL and stored in the CellHotel on the Patchliner (Nanion Technologies GmbH, Munich, Germany) or SyncroPatch 384 (Nanion Technologies GmbH, Munich, Germany), or in an Eppendorf for use on the Port-a-Patch (Nanion Technologies GmbH, Munich, Germany) APC platform, before being dispensed into each well of the NPC-1 (Port-a-Patch), NPC-16 (Patchliner) or NPC-384 (SyncroPatch 384) chip. The standard physiological external solution for all automated patch experiments consisted of (in mM), 140 NaCl 4 KCl, 2 CaCl_2_, 1 MgCl_2_, 10 HEPES, 5 glucose with pH adjusted to 7.4 using NaOH and osmolarity in the range of 295–300 mOsm. The internal solution for measurements of action potentials and voltage-gate potassium channels consisted of (in mM), 110 KF, 10 KCl, 10 NaCl, 1.5 MgCl_2_, 2Na-ATP, 10 EGTA, 10 HEPES with pH adjusted to 7.2 using KOH and osmolarity in the range of 282–288 mOsm. The internal solution for measurement of TTX-resistant Na_V_ currents and P2XR currents consisted of (in mM), 110 CsF, 10 CsCl, 10 NaCl, 10 EGTA, 10 HEPES with pH adjusted to 7.2 with CsOH and osmolarity in the range of 282–288 mOsm. The internal solution for measurement of GABA receptor currents consisted of (in mM), 70 CsF, 60 CsCl, 10 EGTA, 10 HEPES, 2Na-ATP with pH adjusted to 7.2 with CsOH and osmolarity in the range of 282–288 mOsm. Standard patch clamp electrophysiology internal (KF-based) and external solutions were used to record inward Na_V_ and outward K_V_ currents and action potentials, and Na_V_ currents were isolated by using a CsF-based internal solution. Standard voltage and current clamp protocols were used to set holding potential and measure resting membrane potential, and voltage-gated currents and action potentials were evoked with step voltage or current pulses. Cells were only used for analysis if they reached certain QC parameters, R_Seal_ > 300 MOhm, Cm > 2pF, for TTX_r_ currents an additional QC parameter of post-TTX current amplitude > 50 pA following a pre-TTX current amplitude of > 500 pA. Data was acquired using PatchControl 384 (Nanion Technologies GmbH, Munich, Germany) and analyzed using DataControl 384 (Nanion Technologies GmbH, Munich, Germany) on the SyncroPatch 384. Data on the Patchliner and Port-a-Patch was acquired using PatchMaster (HEKA Elektronic, Stuttgart, Germany) and analyzed using IgorPro (Wavemetrics, OR, USA).

## CRediT authorship contribution statement

**Vincent Truong:** Writing – review & editing, Writing – original draft, Methodology, Formal analysis, Data curation, Conceptualization. **Jackson Brougher:** Writing – review & editing, Writing – original draft, Methodology, Data curation, Conceptualization. **Tim Strassmaier:** Writing – review & editing, Writing – original draft, Supervision, Methodology, Formal analysis, Data curation, Conceptualization. **Yi-Ling Lu:** Writing – review & editing, Writing – original draft, Methodology, Formal analysis, Data curation. **Dale George:** Writing – review & editing, Writing – original draft, Data curation, Conceptualization. **Theodore J. Price:** Writing – review & editing, Writing – original draft, Data curation, Conceptualization. **Alison Obergrussberger:** Writing – review & editing, Writing – original draft, Formal analysis, Conceptualization. **Rodolfo J. Haedo:** Writing – review & editing, Writing – original draft, Conceptualization. **Niels Fertig:** Writing – review & editing, Resources, Conceptualization. **Patrick Walsh:** Writing – review & editing, Writing – original draft, Resources, Methodology, Formal analysis, Data curation, Conceptualization.

## Declaration of competing interest

The authors declare the following financial interests/personal relationships which may be considered as potential competing interests: Alison Obergrussberger reports a relationship with Nanion Technologies GmbH that includes: employment. Niels Fertig reports a relationship with Nanion Technologies GmbH that includes: employment. Rodolfo Haedo reports a relationship with Nanion Technologies Inc. that includes: employment. Tim Strassmaier reports a relationship with Nanion Technologies Inc. that includes: employment. Yi-Ling Lu reports a relationship with Nanion Technologies Inc. that includes: employment. Patrick Walsh reports a relationship with Anatomic Incorporated that includes: employment. Vince Truong reports a relationship with Anatomic Incorporated that includes: employment. Co-author Theodore J. Price serves as the Editor-in-Chief of the Neurobiology of Pain. Given his role as Editor-in-Chief of the Neurobiology of Pain, Theodore J. Price had no involvement in the peer review of this article and had no access to information regarding its peer review. Full responsibility for the editorial process for this article was delegated to another journal editor. If there are other authors, they declare that they have no known competing financial interests or personal relationships that could have appeared to influence the work reported in this paper.

## Data Availability

Sequence data is available in GEO, accession numbers GSE275412 (bulk) and GSE275413 (single cell). A searchable version of the bulk RNAseq data is also made available at https://anatomicincorporated.shinyapps.io/realdrgene/.

## Appendix A Supplementary data

Supplementary Data 1 The supplementary material includes a table comparing neuron differentiation strategies; extra heatmap analyses for ion channel expression along with expression of neuropeptides, GPCRs and cell adhesion molecules, and examples of action potentials before and after the application of TTX in hiSNs.
