# Supplementary material for: Transcriptomic analysis and high throughput functional characterization of human induced pluripotent stem cell derived sensory neurons
Source: Neurobiol Pain. 2026 May 1;20:100216. doi: 10.1016/j.ynpai.2026.100216 (PMC13156722; doi:10.1016/j.ynpai.2026.100216)
Supplement: Supplementary Data 1 — The supplementary material includes a table comparing neuron differentiation strategies; extra heatmap analyses for ion channel expression along with expression of neuropeptides, GPCRs and cell adhesion molecules, and examples of action potentials before and after the application of TTX in hiSNs. [file mmc1.pptx]

## Slide 1
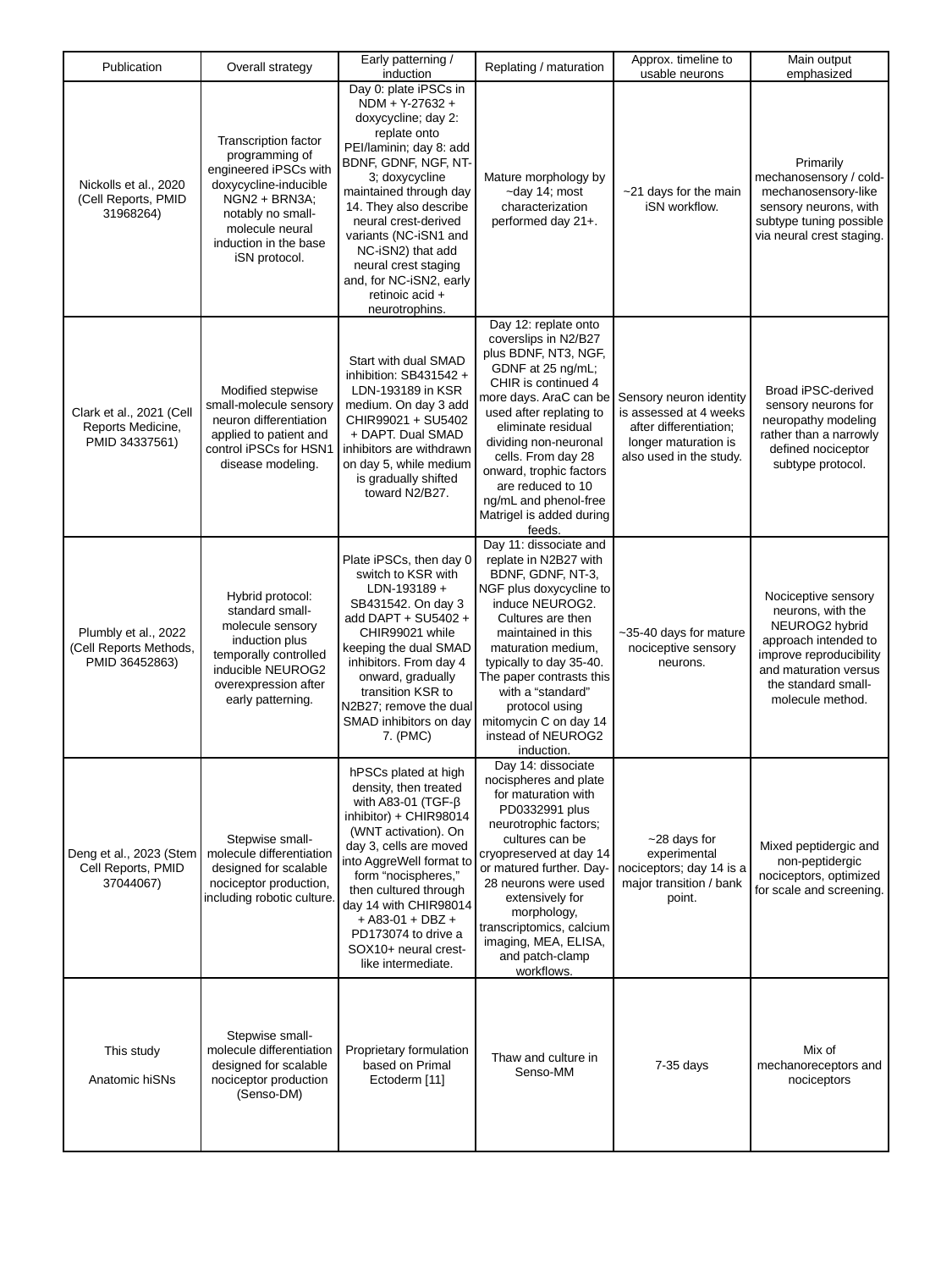

| Publication | Overall strategy | Early patterning / induction | Replating / maturation | Approx. timeline to usable neurons | Main output emphasized |
| --- | --- | --- | --- | --- | --- |
| Nickolls et al., 2020 (Cell Reports, PMID 31968264) | Transcription factor programming of engineered iPSCs with doxycycline-inducible NGN2 + BRN3A; notably no small-molecule neural induction in the base iSN protocol. | Day 0: plate iPSCs in NDM + Y-27632 + doxycycline; day 2: replate onto PEI/laminin; day 8: add BDNF, GDNF, NGF, NT-3; doxycycline maintained through day 14. They also describe neural crest-derived variants (NC-iSN1 and NC-iSN2) that add neural crest staging and, for NC-iSN2, early retinoic acid + neurotrophins. | Mature morphology by ~day 14; most characterization performed day 21+. | ~21 days for the main iSN workflow. | Primarily mechanosensory / cold-mechanosensory-like sensory neurons, with subtype tuning possible via neural crest staging. |
| Clark et al., 2021 (Cell Reports Medicine, PMID 34337561) | Modified stepwise small-molecule sensory neuron differentiation applied to patient and control iPSCs for HSN1 disease modeling. | Start with dual SMAD inhibition: SB431542 + LDN-193189 in KSR medium. On day 3 add CHIR99021 + SU5402 + DAPT. Dual SMAD inhibitors are withdrawn on day 5, while medium is gradually shifted toward N2/B27. | Day 12: replate onto coverslips in N2/B27 plus BDNF, NT3, NGF, GDNF at 25 ng/mL; CHIR is continued 4 more days. AraC can be used after replating to eliminate residual dividing non-neuronal cells. From day 28 onward, trophic factors are reduced to 10 ng/mL and phenol-free Matrigel is added during feeds. | Sensory neuron identity is assessed at 4 weeks after differentiation; longer maturation is also used in the study. | Broad iPSC-derived sensory neurons for neuropathy modeling rather than a narrowly defined nociceptor subtype protocol. |
| Plumbly et al., 2022 (Cell Reports Methods, PMID 36452863) | Hybrid protocol: standard small-molecule sensory induction plus temporally controlled inducible NEUROG2 overexpression after early patterning. | Plate iPSCs, then day 0 switch to KSR with LDN-193189 + SB431542. On day 3 add DAPT + SU5402 + CHIR99021 while keeping the dual SMAD inhibitors. From day 4 onward, gradually transition KSR to N2B27; remove the dual SMAD inhibitors on day 7. (PMC) | Day 11: dissociate and replate in N2B27 with BDNF, GDNF, NT-3, NGF plus doxycycline to induce NEUROG2. Cultures are then maintained in this maturation medium, typically to day 35-40. The paper contrasts this with a “standard” protocol using mitomycin C on day 14 instead of NEUROG2 induction. | ~35-40 days for mature nociceptive sensory neurons. | Nociceptive sensory neurons, with the NEUROG2 hybrid approach intended to improve reproducibility and maturation versus the standard small-molecule method. |
| Deng et al., 2023 (Stem Cell Reports, PMID 37044067) | Stepwise small-molecule differentiation designed for scalable nociceptor production, including robotic culture. | hPSCs plated at high density, then treated with A83-01 (TGF-β inhibitor) + CHIR98014 (WNT activation). On day 3, cells are moved into AggreWell format to form “nocispheres,” then cultured through day 14 with CHIR98014 + A83-01 + DBZ + PD173074 to drive a SOX10+ neural crest-like intermediate. | Day 14: dissociate nocispheres and plate for maturation with PD0332991 plus neurotrophic factors; cultures can be cryopreserved at day 14 or matured further. Day-28 neurons were used extensively for morphology, transcriptomics, calcium imaging, MEA, ELISA, and patch-clamp workflows. | ~28 days for experimental nociceptors; day 14 is a major transition / bank point. | Mixed peptidergic and non-peptidergic nociceptors, optimized for scale and screening. |
| This study Anatomic hiSNs | Stepwise small-molecule differentiation designed for scalable nociceptor production (Senso-DM) | Proprietary formulation based on Primal Ectoderm [11] | Thaw and culture in Senso-MM | 7-35 days | Mix of mechanoreceptors and nociceptors |

## Slide 2
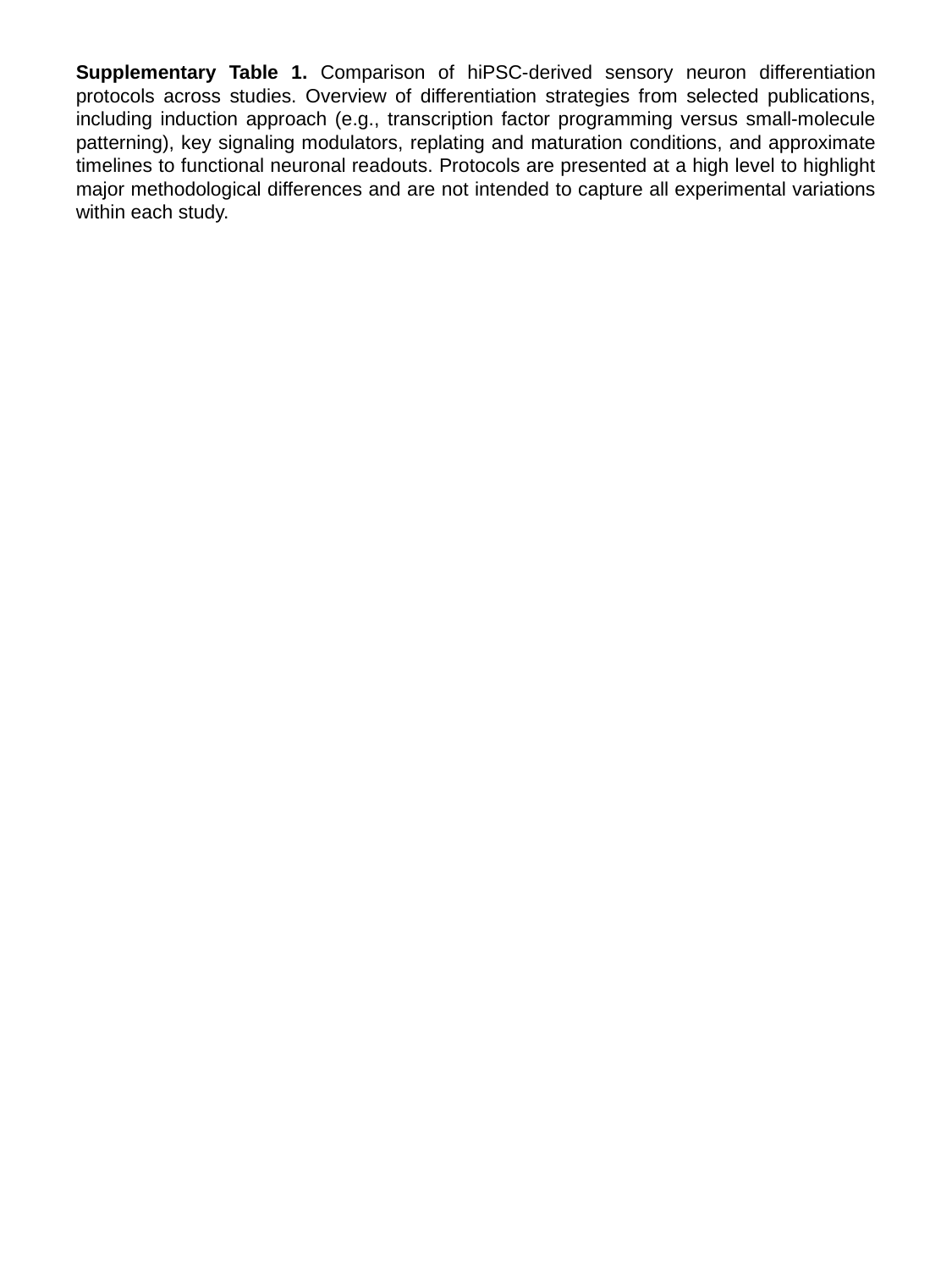

Supplementary Table 1. Comparison of hiPSC-derived sensory neuron differentiation protocols across studies. Overview of differentiation strategies from selected publications, including induction approach (e.g., transcription factor programming versus small-molecule patterning), key signaling modulators, replating and maturation conditions, and approximate timelines to functional neuronal readouts. Protocols are presented at a high level to highlight major methodological differences and are not intended to capture all experimental variations within each study.

## Slide 3
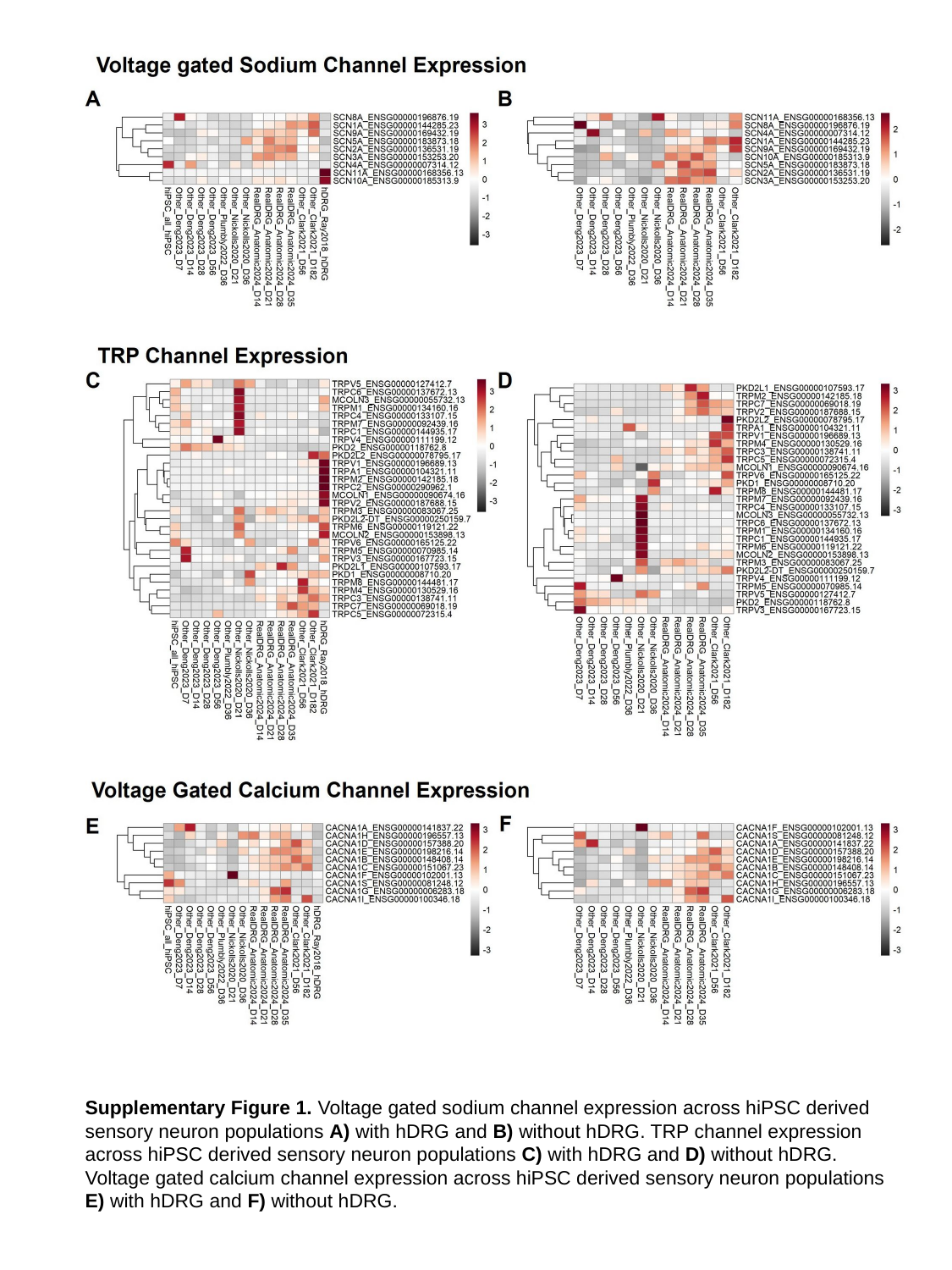

Supplementary Figure 1. Voltage gated sodium channel expression across hiPSC derived sensory neuron populations A) with hDRG and B) without hDRG. TRP channel expression across hiPSC derived sensory neuron populations C) with hDRG and D) without hDRG. Voltage gated calcium channel expression across hiPSC derived sensory neuron populations E) with hDRG and F) without hDRG.

## Slide 4
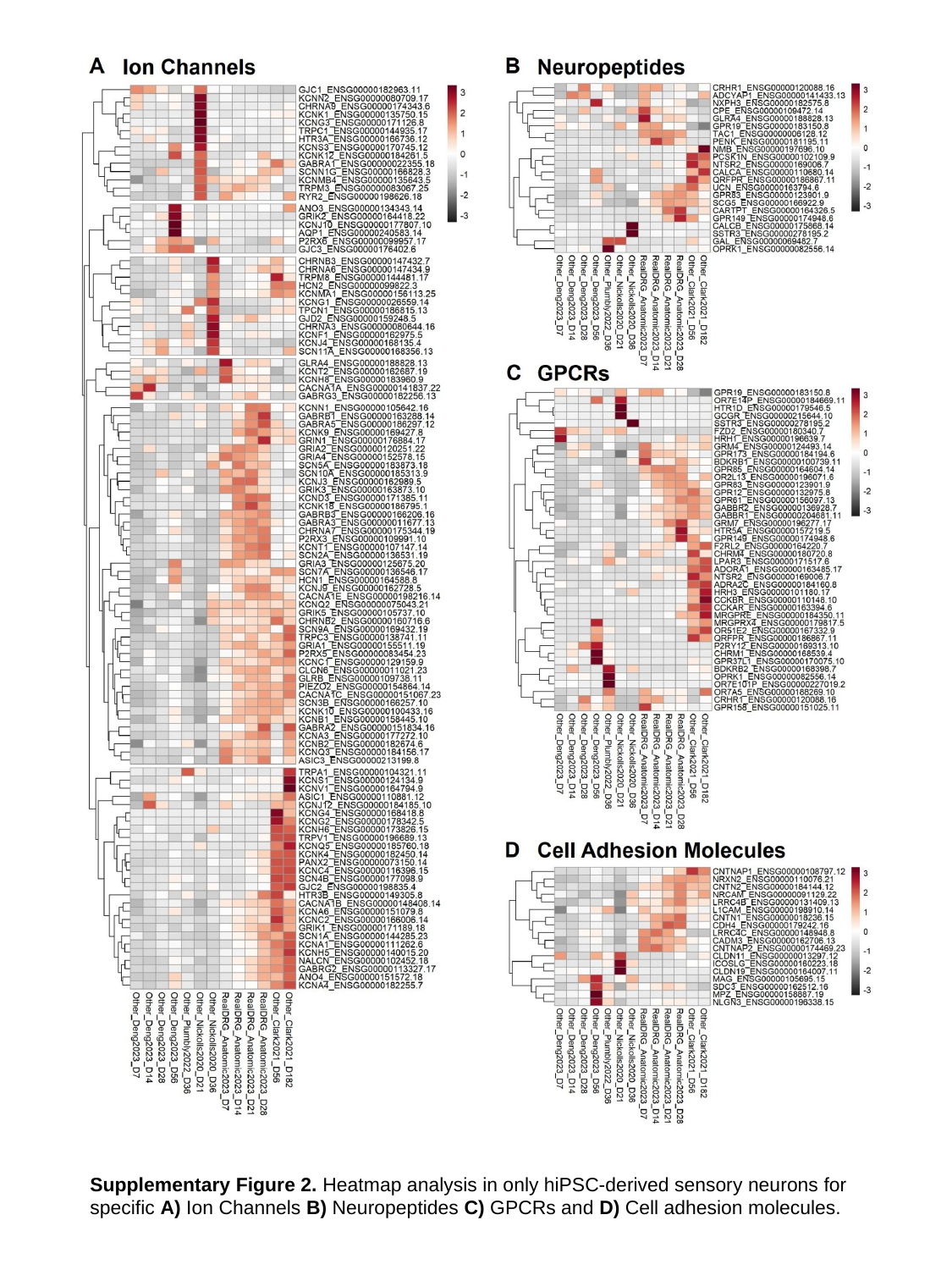

Supplementary Figure 2. Heatmap analysis in only hiPSC-derived sensory neurons for specific A) Ion Channels B) Neuropeptides C) GPCRs and D) Cell adhesion molecules.

## Slide 5
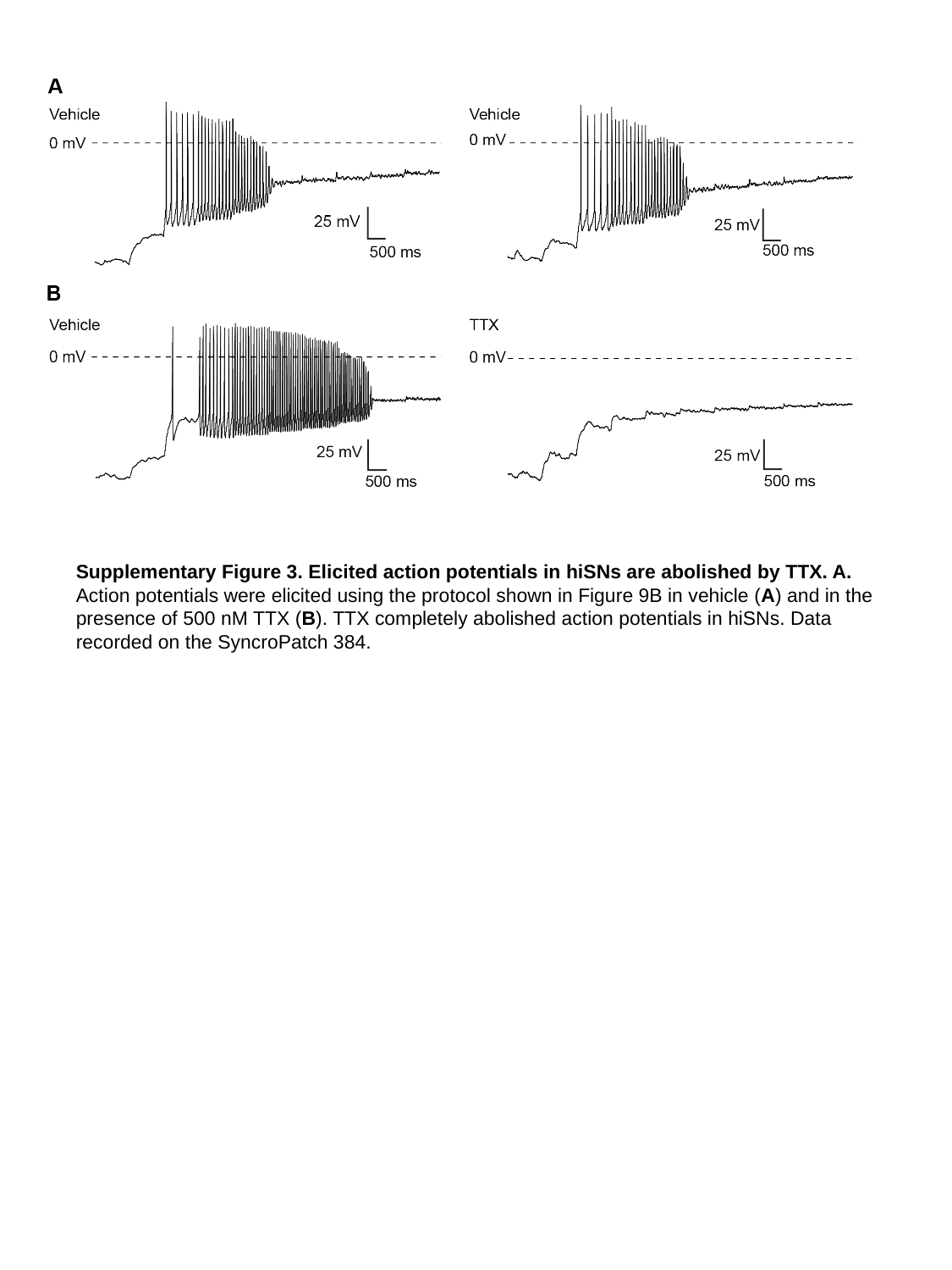

Supplementary Figure 3. Elicited action potentials in hiSNs are abolished by TTX. A. Action potentials were elicited using the protocol shown in Figure 9B in vehicle (A) and in the presence of 500 nM TTX (B). TTX completely abolished action potentials in hiSNs. Data recorded on the SyncroPatch 384.
